# Comprehensive analysis of regulation of DNA methyltransferase isoforms in human breast tumors

**DOI:** 10.1007/s00432-021-03519-4

**Published:** 2021-02-18

**Authors:** Mangala Hegde, Manjunath B. Joshi

**Affiliations:** Manipal School of Life Sciences, Manipal Academy of Higher Education, Planetarium Complex, Manipal, 576104 India

**Keywords:** Breast cancer, Epigenetics, DNA methylation, DNA methyltransferases

## Abstract

Significant reprogramming of epigenome is widely described during pathogenesis of breast cancer. Transformation of normal cell to hyperplastic cell and to neoplastic phenotype is associated with aberrant DNA (de)methylation, which, through promoter and enhancer methylation changes, activates oncogenes and silence tumor suppressor genes in variety of tumors including breast. DNA methylation, one of the major epigenetic mechanisms is catalyzed by evolutionarily conserved isoforms namely, DNMT1, DNMT3A and DNMT3B in humans. Over the years, studies have demonstrated intricate and complex regulation of DNMT isoforms at transcriptional, translational and post-translational levels. The recent findings of allosteric regulation of DNMT isoforms and regulation by other interacting chromatin modifying proteins emphasizes functional integrity and their contribution for the development of breast cancer and progression. DNMT isoforms are regulated by several intrinsic and extrinsic parameters. In the present review, we have extensively performed bioinformatics analysis of expression of DNMT isoforms along with their transcriptional and post-transcriptional regulators such as transcription factors, interacting proteins, hormones, cytokines and dietary elements along with their significance during pathogenesis of breast tumors. Our review manuscript provides a comprehensive understanding of key factors regulating DNMT isoforms in breast tumor pathology and documents unsolved issues.

## Introduction

Neoplasia are the uncontrolled growth of cells as a consequence of disrupted gene expression and associated signaling pathways as a consequence of genetic or epigenetic changes (Hanahan and Weinberg 2000). The term ‘epigenetics’ refers to heritable covalent modifications of chromatin components which, by transforming the chromatin organization, affect accessibility of DNA for the regulatory and transcription factors without affecting the basic nucleotide sequence (Egger et al. 2004). The epigenetic machinery regulates gene expression by (a) DNA methylation; (b) post-translational modifications of histones, and (c) non-coding RNAs. Methylation of DNA is a vital process during development, cellular differentiation and tissue homeostasis (Feil and Fraga 2012). DNA methylation is a process, where methyl group is covalently attached to C-5 of the cytosine residue and catalyzed by evolutionarily conserved isoforms of DNA methyl transferases (DNMTs). The mechanism of DNA methylation is widely associated with various physiological processes such as X chromosome inactivation, chromosome stability, genomic imprinting, tissue specific gene expression, repression of transposable elements and aging (Bernstein et al. 2007). Besides various genetic alterations such as mutations, loss of heterozygosity and inducing copy number variations, cancer cells harbor global epigenetic alterations leading to growth and metastasis demonstrating the complex interplay between genetic and epigenetic mechanisms in (dys)regulation of gene expression (Sadikovic et al. 2008). Recent advances in high throughput DNA sequencing and single cell DNA methylation analysis have revealed existence of distinct epigenetic signatures in variety of cancer types and the extent of epigenetic changes is correlated with tumor stage and type (Fernandez et al. 2012). The functions affecting DNMT isoforms including mutations are correlated with the biological characteristics of malignancy and enhance the proliferation, migration, invasion, stemness, epithelial mesenchymal transition and metastasis of tumor cells (Dawson and Kouzarides 2012). The widespread epigenetic defects including DNA methylation in breast tumors instigated us to revisit the regulation of DNMT isoforms in these pathological conditions. Hence, the present review aimed to assimilate the existing knowledge of genetic and epigenetic regulation of DNMT isoforms in breast tumors along with functional consequences.

## (Dys)regulation of DNA methylation during tumorigenesis

Based on the structures, DNMT isoforms are classified into DNMT1, DNMT2 and DNMT3 family. DNMT1, DNMT3A and DNMT3B isoforms expressed in human tissues are encoded by distinct genes *DNMT1 (DNMT, AIM, MCMT, CXXC9, HSNE1, ADCADN), DNMT3A (DNMT3A2, TBRS, HESJAS), DNMT3B (ICF, ICF1)* localized on chromosome 19, 2 and 20 respectively. Three major DNA methyl transferases are involved in initiation and maintenance of DNA methylation patterns in humans: (a) DNMT1 (maintenance methyl transferase), has a strong predilection for hemi-methylated CpG dinucleotides, consequently methylates the newly synthesized DNA strand considering the methylation in the complementary strand as gold standard; (b) DNMT2 is shown to methylate tRNA anticodon loop and DNA methylation activity of DNMT2 is reported to be low or absent; (c) DNMT3 (de novo methyl transferases) isoforms are involved in de novo methylation and non-CpG methylation (Ramsahoye et al. 2000; Jones and Baylin 2002; Laird 2003; Hermann et al. 2003; Goll et al. 2006). DNMT3 consists of three subtypes: DNMT3A, DNMT3B and DNMT3-like protein (DNMT3L). DNMT3A and DNMT3B possess catalytic activities and are regulated by DNMT3L (Okano et al. 1999; Hu et al. 2008).

Alteration of DNA methylation pattern is closely associated with the initiation and progression of tumors. Rauscher et al., 2015 showed that the frequent DNA methylation alteration in promoter regions, introns, far upstream regions, LINE-1 and satellite 2 DNA repeats were associated with the breast cancer development (Rauscher et al. 2015). Increased methylation in promoter CpG islands of specifically tumor suppressor genes including *p16*^*INK4A*^*, p15*^*INK4A*^*, p53, p73, TIMP-3, BRCA1, PLCD1, PCDH17, RASSF1A, HIN-1, FOXD3, MLH1, MSH2, ERCC1, RUNX3, GATA-4* and* GATA-5* are frequently reported in several cancers such as hematological malignancies and tumors of lung, colon, breast, neurological, liver, nasopharyngeal, ovarian and endometrium (Kang et al. 2001b; Feng et al. 2010; Quintás-Cardama et al. 2012; Xing et al. 2013; Zhu et al. 2015; Cosgrove et al. 2017; Maleva Kostovska et al. 2018; Hentze et al. 2019; Xu et al. 2019). Global hypomethylation of DNA at various genomic locations including CpG-poor promoters, repeat sequences and retrotransposons results in the overexpression of proto-oncogenes and growth factors attributes to hallmarks of cancer. For instance, hypomethylation of uPA resulting in its overexpression is correlated with progression of breast, prostate and brain tumors (Pakneshan et al. 2005; Kandenwein et al. 2011). Several studies have shown that the loss of imprinting of *IGF-2* due to hypomethylation leads to uncontrolled proliferation of tumor cells (Leick et al. 2011). Hypomethylation of Alu repeats in the intronic region of *TGFB2* and region overlapping the CpG island of the *PRDM16* exon has been observed in tumor cell lines (Irizarry et al. 2009). Joo et al. (2018) showed that heritable DNA methylation pattern is a major contributor for the breast cancer risk in multiple case breast cancer families with no known genetic mutation (Joo et al. 2018).

Overexpression of DNMT1, DNMT3A and DNMT3B at both transcriptional and translational levels which, in turn leads to reduced expression of tumor suppressor genes has been reported in several malignancies including colorectal, lung cancer, glioblastomas, hematological malignancies, prostate and breast (De Marzo et al. 1999; Mizuno et al. 2001; Girault et al. 2003; McCabe et al. 2005; Lin et al. 2007; Lorente et al. 2009; Gravina et al. 2013; Yu et al. 2015; San José-Enériz et al. 2017). Enhanced expression of DNMT1 in tumor tissues is a suggestive for increased aggressiveness of the disease and poor prognosis. Rhee et al. (2002) showed that disruption of either DNMT1 or DNMT3B resulted in partial methylation and simultaneous disruption of DNMT1 and DNMT3B resulted in global hypomethylation and reactivation of tumor suppressor genes in both in vitro and in vivo colorectal cancer models leading to reduced proliferation and tumor growth (Rhee et al. 2002). This indicated that coordinated activity of DNMT1 and DNMT3B might be essential for neoplastic transformation. Furthermore, Xiong et al. (2005) demonstrated that endometrioid cancers frequently showing hypermethylation in the promoters of tumor suppressor genes over expressed DNMT1 and DNMT3B and serous endometrial cancers developed due to P53 mutation, loss of heterozygosity and aneuploidy showed substantial reduction in DNMT1 and DNMT3B levels than controls (Xiong et al. 2005). Furthermore, authors also showed that levels of DNMT1 and DNMT3B were higher in poorly differentiated tumorigenic cell lines such as AN3, KLE, RL-95, HEC1A and HEC1B- compared to differentiated non-tumorigenic Ishikawa cell lines (Xiong et al. 2005). Approximately, 30% of breast cancer patients showed overexpression of DNMT3B and 3–5% showed overexpression of DNMT1 and DNMT3A. Roll et al. (2008) showed that over expression of DNMT3B in breast cancer was strongly correlated with total DNMT1 activity (Roll et al. 2008). Furthermore, studies have shown that global DNA methylation and promoter CpG hypermethylation have been reported to occur simultaneously as independent mechanisms during breast tumorigenesis and at various cancer stages. However, it has been proposed that global DNA hypomethylation may be a process to occur at the later stages since increased degree of global hypomethylation of DNA has been noted with increase in lesion progression. On the other hand, promoter hypermethylation may be an early event during breast tumor development (Tan et al. 2013). Taken together, these data suggested that expression of DNMTs is highly regulated in the tissue and regulators of DNMT expression and activity might play an important role in the dysfunction of DNA methylation machinery.

## DNA methylation in breast tumors

Paradoxical DNA methylation changes have been observed in breast cancer: regional hypermethylation of specific genes and global hypomethylation. Regional hypermethylation silences genes involved in cell cycle and growth regulation leading to uncontrolled growth of cells, whereas hypomethylation is a requisite for metastasis (Steeg et al. 2003). The semiquantitative methylation changes through mass spectrometry-based analysis and CpG microarray data have shown that regional hypermethylation signatures in breast cancer have unique combination of CpG islands which is correlated with stage of the disease and is proposed for exploring as diagnostic and prognostic marker(s). Several crucial genes such as *p16, BRCA1, MLH1, HMSH2, ESR1, ESR2, RARB, CDH9, PRAC2, TDR10, APC, GSTP1, BIN1, BMP6, CST6, DKK3, RASSF1A, HOXD13, SFN, PITX2, SFRP1, CD3D, CD6, LAX1, UBE2C, TOPBP1* and *TIMP3* involved in cell cycle, DNA repair and adhesion were hypermethylated in breast tumors (Radpour et al. 2009; Győrffy et al. 2016). Teschendroff et al. (2016) analyzed 397 breast tumor samples including adjacent normal tissue and demonstrated that differential methylation marks which accounted for 20–30% changes in beta values. Furthermore, the authors showed that these epigenetic signatures are heterogenous and epigenetic changes in adjacent stromal cells were responsible for the aggressiveness of tumor progression (Teschendorff et al. 2016).

**Fig. 1:**
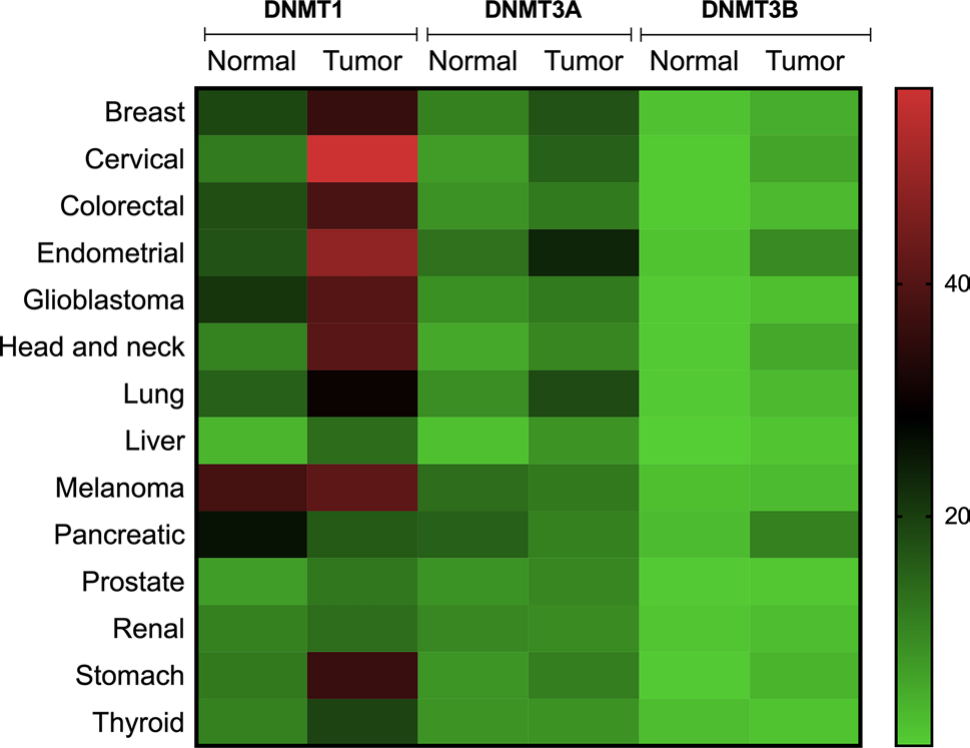
Differential expression of DNMT isoforms in various cancers. The gene expression of DNMT1, 3A and 3B based on RNA sequence data (transcript per million) for various cancers- breast cancer (normal, *n* = 114; tumor, *n* = 1097), glioblastoma (normal, *n* = 5; tumor, *n* = 156), thyroid cancer (normal, *n* = 59; tumor, *n* = 505), lung cancer (normal, *n* = 59; tumor, *n* = 515), cervical cancer (normal, *n* = 3; tumor, *n* = 305), colon cancer (normal, *n* = 41; tumor, *n* = 286), endometrial cancer (normal, *n* = 35; tumor, *n* = 546), head and neck (normal, *n* = 44; tumor, *n* = 520), liver cancer (normal, *n* = 50; tumor, *n* = 371), melanoma (normal, *n* = 1; tumor, *n* = 104), prostate (normal, *n* = 592; tumor, *n* = 497), pancreatic (normal, *n* = 4; tumor, *n* = 178), renal cancer (normal, *n* = 72; tumor, *n* = 533), stomach cancer (normal, *n* = 34; tumor, *n* = 415), testis cancer (normal, *n* = 59; tumor, *n* = 505), were downloaded from TCGA database and heatmap is plotted

**Fig. 2:**
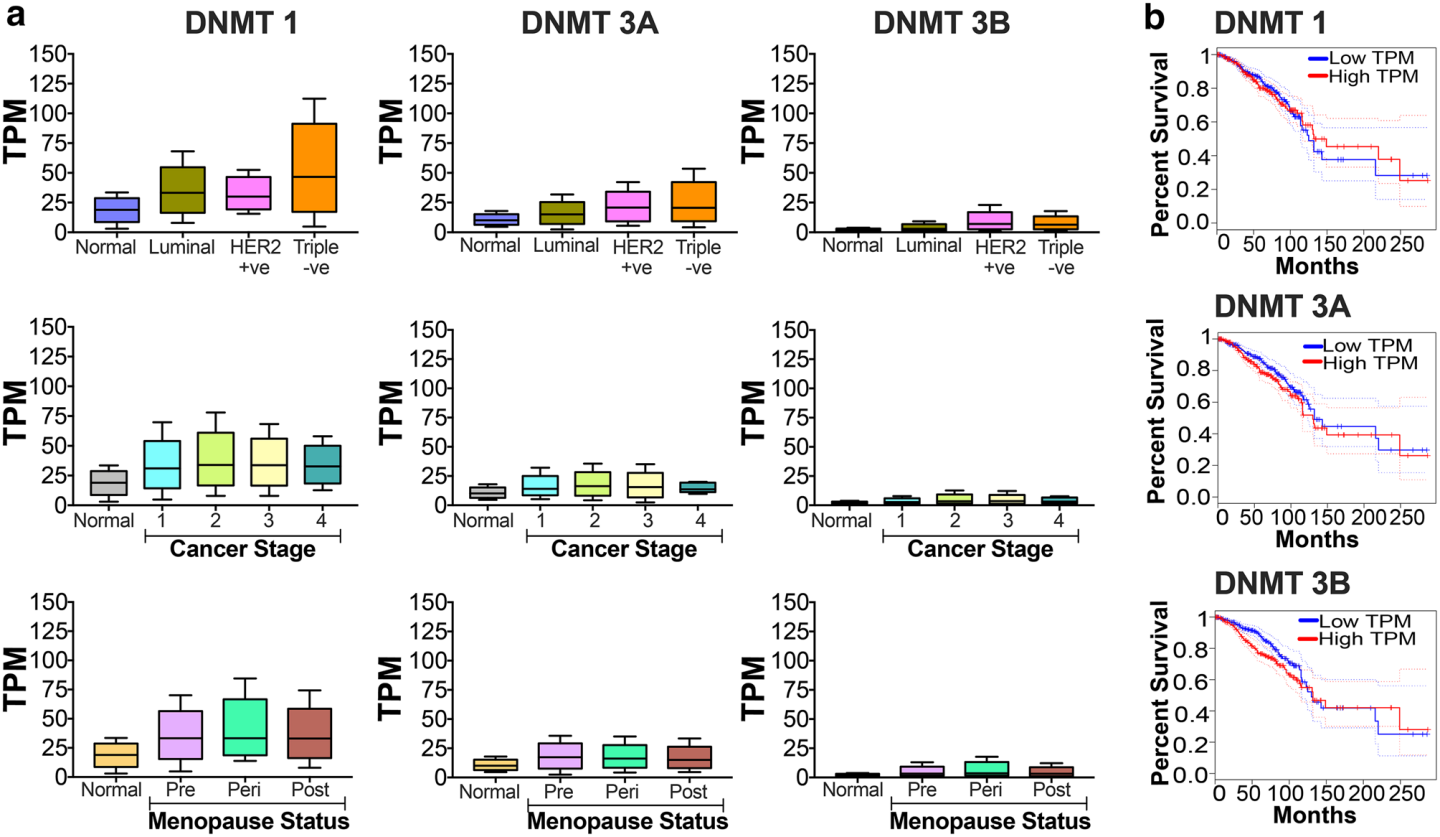
DNMTs levels are altered in breast cancer: Gene expression data from the TCGA was extracted. **a** The levels of DNMT1, 3A and 3B in major subclasses of breast cancer- normal (*n* = 114), luminal (*n* = 566), Her2 positive (*n* = 37), triple negative (*n* = 116); different stages of cancer- normal (*n* = 114), stage 1 (*n* = 183), stage 2 (*n* = 615), stage 3 (*n* = 247), stage 4 (*n* = 20) and levels in pre- (*n* = 230), peri- (*n* = 37) and post- (*n* = 700) menopausal women are plotted. **b** Survival analysis for DNMT1, DNMT3A and DNMT3B are carried out and data is shown. Red-High expression level (*n* = 810), Blue-Low expression level (*n* = 271)

TCGA-based bioinformatic analysis revealed that expression levels of DNMT1, 3A and 3B altered in several cancers including breast cancers (Fig. 1). Over the years, various independent studies have demonstrated significant role of DNMT isoforms in breast tumors and hence, promoted us to look at changes in expression levels of DNMT isoforms across different types of breast cancers, at different stages and considering menopause status (Fig. 2a). We mined TCGA database using http://ualcan.path.uab.edu/index.html. The expression levels of DNMT1 and DNMT3A showed highest expression levels in triple negative breast cancer patients, where previous studies have observed hypermethylation of tumor suppressor genes. The DNMT1 and DNMT3A levels were down regulated in the fourth stage and interestingly, correlated with global hypomethylation as a marked signature of metastasis. Expression pattern of DNMT isoform did not vary significantly among pre-, peri- and post-menopause status. However, tumor tissues showed significantly increased levels compared to normal tissues. DNMT3B transcripts were low compared to that of DNMT1 and 3A in different types of breast cancers and stages (Fig. 2a). Furthermore, the survival analysis revealed that increased levels of DNMT3B significantly correlated with the decreased overall survival rate (*p* < 0.01) in breast cancer patients. However, marginal increased levels of DNMT1 (*p* > 0.05) and DNMT3A (*p* > 0.05) did not significantly reflected on survival rate (Fig. 2b). This suggested DNMT isoforms and their target genes might serve as good indicators of prognosis in breast cancers and hence we looked at status of various transcriptional regulators of DNMT isoforms in breast tumors.

## Status of regulatory proteins influencing expression of DNMT isoforms in breast tumor tissues

FANTOM5 consortium includes single molecule CAGE profiles across 573 human samples covering major mammalian cell steady states. The data contains complete profiles of 250 different cancer cell lines and 152 human post-mortem tissue samples. Zenbu genome browser is a web-based interactive dynamic CAGE and TSS (transcription start site) exploration platform which enables to survey TSS activity within defined genomic region with user selectable alignment (Severin et al. 2014).

**Fig. 3:**
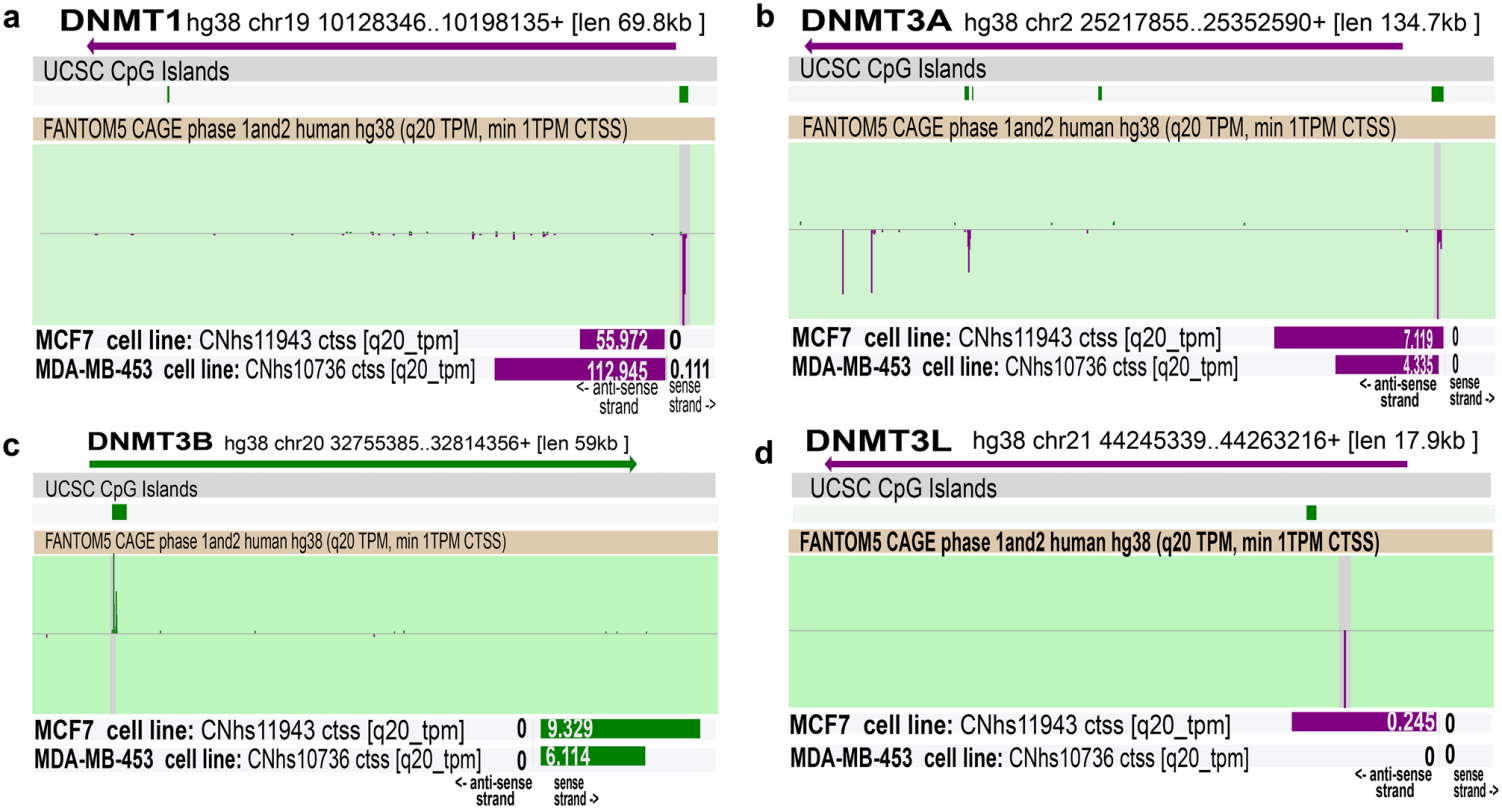
DNMT isoforms locus, TSS and TSS activity in breast cancer. Images are the screen shot of ZENBU browser showing the locus of DNMT1(**a**), DNMT3A (**b**), DNMT3B (**c**) and DNMT3L (**d**). The track UCSC CpG islands shows number and locus of CpG islands and FANTOM 5 CAGE Phase 1 and 2 track represents histogram of CAGE tag counts across the entire dataset. The last track shows the TSS activity in breast carcinoma cell line MCF 7 and breast carcinoma cell line MDA-MB-453. Purple, antisense strand; Green, sense strand

Our Zenbu analysis of DNMT isoforms revealed different TSS for single isoforms across the genome with varied activities. The highest and lowest active TSS sites are shown in Fig. 3. The TSS activity of DNMT1 (Fig. 3a) and DNMT3A (Fig. 3b) were highest in triple negative MDA-MB-453 among breast tumor cell lines. Activity of DNMT 3B (Fig. 3c) TSS activity was highest in MCF-7 cell lines compared to MDA-MB-453 cell line. However, the TSS activity of DNMT3L was found nil (0.0) in both breast cancer cell lines (Fig. 3d).

### Transcriptional regulation

**Fig. 4:**
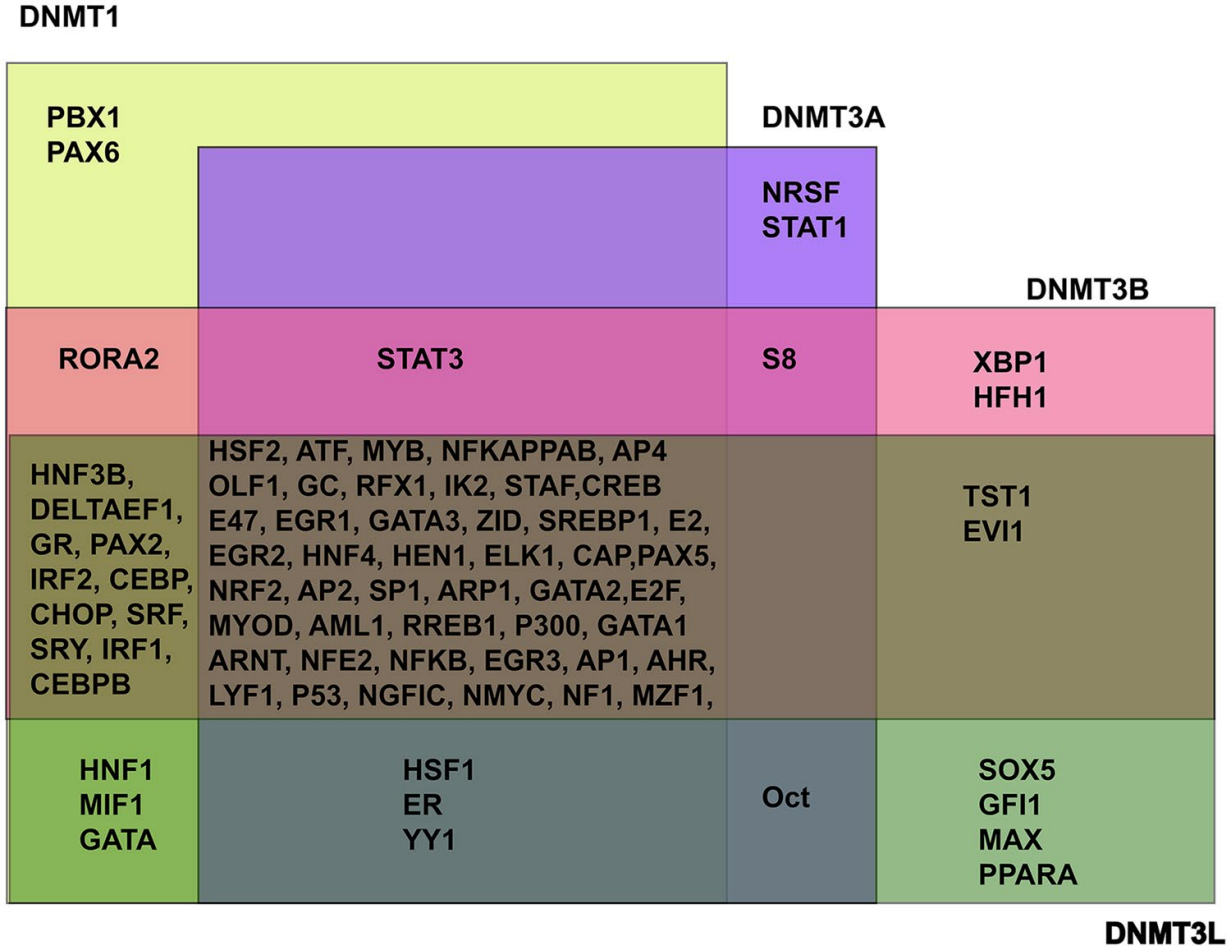
Redundancy in transcription factors (TFs) interaction with DNMT isoforms. In vitro experiments such as transcriptional factor arrays, CHIP assays, recombinant DNMT1, 3A, 3B and 3L constructions have revealed that certain TFs can potentially interact with all the isoforms or few isoforms or only with one specific isoform. The TFs binding to − 500 to + 200 region of DNMT isoforms were retrieved from TF-binding input tool and validated using ContraV3 tool and UCSC genome browser. Fluorescent light green, TFs binding only to DNMT1; Purple, TFs binding only with DNMT3B; Pink, TFs interact with DNMT3B; Olive green, TFs bind to DNMT3L only; Overlaps regions shows those TFs which bind to more than one isoform

Our bioinformatic analysis indicated that DNMT isoforms interact with several transcription factors including p53, SP1, SP3, E2, p300, E47. Transcription factors such as PBX1 and PAX6 were found to interact with only DNMT1, NRSF1, STAT1 with DNMT3A, XBP1 and HFH1 with DNMT3B and SOX5, GFI1, MAX, PPARA with only DNMT3L. However, certain other transcription factors such as HASF2, ATF, MYB, NFKAPPAB, AP4, OLF1, GC, RFX1, IK2, STAF, CREB, E47, EGR1, GATA3, ZID, SREBP1, E2, EGR2, HNF4, HEN1, ELK1, CAP, PAX5, NRF2, AP2, SP1, ARP1, GATA2, E2F, MYOD, AML1, RREB1, P300, GATA1, ARNT, NFE2, NFKB, EGR3, AP1, AHR, LYF1, P53, NGFIC, NMYC, NF1 and MZF1 shown to be bound to all the four DNMT isoforms. The redundancy in binding of these transcription factors is represented in Fig. 4.

### Transcriptional activation

#### SP1- and SP3-mediated transcriptional activation of DNMT isoforms

**Fig. 5:**
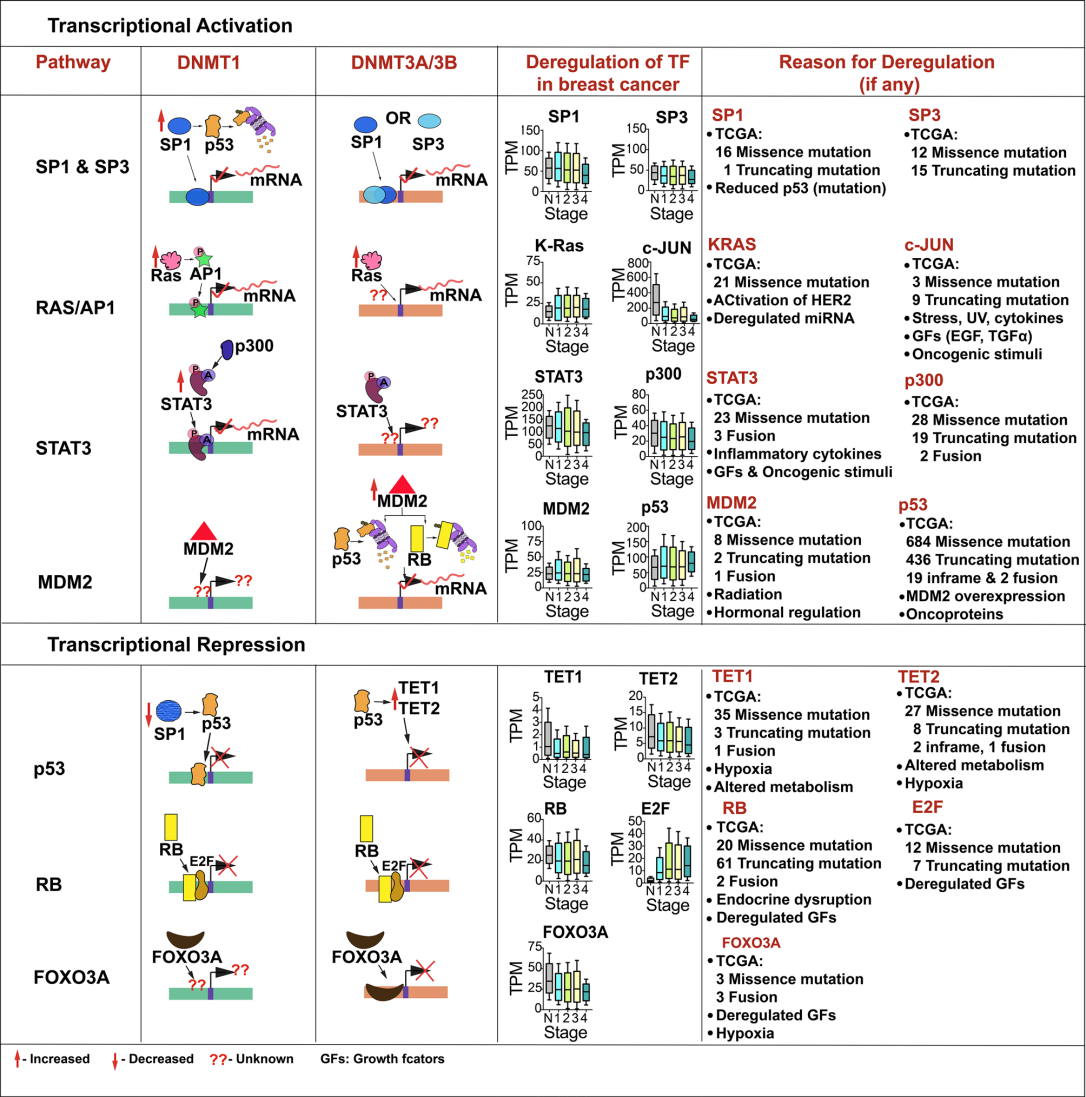
Transcriptional regulation of DNMT isoforms. Regulation of transcriptional activation and repression of DNMT1, DNMT3A and DNMT3B by different pathways and alteration of these factors in different stages of breast cancer-normal (*n* = 114), stage 1 (*n* = 183), stage 2 (*n* = 615), stage 3 (*n* = 247), stage 4 (*n* = 20), the mechanism of deregulation of these factors are shown. *SP1* specificity protein 1, *SP3* specificity protein, *K-RAS* Kristen rat sarcoma viral oncoprotein, *AP1* Activator protein 1, *STAT3* signal transducer and activator of transcription, *p300* E1A-binding protein p300, *MDM2* Mouse double minute 2, *p53* Tumor protein p53, *TET1* ten-eleven translocation 1, *TET2* 10–11 translocation 2, *RB*, Retinoblastoma susceptibility protein, *E2F* PRB-binding protein E2F, *FOXO3A* Forkhead box O3

The SP family transcription factors belongs to conserved zinc finger DNA-binding domain proteins that recognize the GC- rich box (GGGCGGG) and GT rich box (GGTGTGGGG). These factors are important for the expression of different housekeeping genes and genes which are deficient of TATA- or CAAT-boxes in their proximal promoters (Hagen et al. 1992). Several SP proteins have been identified (SP1–SP8) and among these SP1 and SP3 are ubiquitously expressed. SP1 is a transcription activator and SP3 acts as either activator or repressor depending upon the context of either promoter region and cell type (Bouwman and Philipsen 2002). Earlier studies have shown that stoichiometric ratio of SP1 and p53 is required for physical interaction with regulatory element for DNMT1 transcription (Lin et al. 2010b). Total of three putative SP1-binding sites identified on DNMT1 promoter region and one among these binding sites (+ 7 to  + 20) being proximal to binding site of p53 (+ 30 to  + 56). At low levels, SP1 interacts with p53 and represses DNMT1 expression and at higher levels, SP1 targets p53 to proteasomal degradation via MDM2-mediated ubiquitination and directly binds to *DNMT1* promoter to initiate transcription (Lin et al. 2010b). The *cis*-element in *DNMT1* promoter located between -147 to -161 was shown activated by SP1 and SP3 independently of each other and p300 was co-activator for SP3-mediated activation (Kishikawa et al. 2002). Studies have also shown that SP1 and SP3 also acts as the transcriptional activators of DNMT3A and DNMT3B. Minimal promoter regions of both DNMT3A and DNMT3B contain SP1-binding site at − 99 to − 87 and − 100 to − 92 respectively. Overexpression of these SP proteins and site directed mutagenesis in the binding sites indicated that DNMT3A and DNMT3B promoter activities are largely dependent on SP1 and SP3-binding sites (Jinawath et al. 2005). TCGA analysis showed that expression of SP1 transcripts were significantly reduced in breast tumor tissues and SP3 RNA levels were significantly decreased in patients with stage IV disease indicating aberrant expression of SP1 and SP3 might be responsible for DNA methylation changes during breast tumorigenesis (Fig. 5).

#### Involvement of Ras/AP-1 pathway in regulation of DNMT isoforms

Ras superfamily GTPases are the key regulators of cell proliferation, contraction, intracellular asymmetry, cell shape, apoptosis, single and coordinated cell migration. Elevation of Ras-signal has been shown to play an important role in epigenetic silencing of several genes in human tumors (Patra 2008). DNMT1 promoter harbors three c-Jun dependent enhancer regions downstream to P1 and upstream to P2 and P4 and number of AP-1-binding sites in the promoter region which explains the control of Ras signaling pathway on DNMT1 regulation (MacLeod et al. 1995). Aberrant expression of Ras downstream effectors in breast cancer are documented and has been explored as therapeutic target. Pakneshan et al (2005) showed that downregulation of uPA (urokinase type plasminogen activator) in highly metastatic breast cancer cell line MDA-MB-231 via up regulation of Ras-mediated DNMT1 leading to uncontrolled cell growth (Pakneshan et al. 2005). Furthermore, authors showed that the promoter methylation of uPA was reversed in MDA-MB-231 cell lines upon the treatment of 5^′^-azacytidine (Pakneshan et al. 2005). Elangovan et al (2013) demonstrated that *SLC5A8* a putative tumor suppressor gene is inactivated due to promoter hypermethylation via HRas induced expression of DNMT1 leading to tumorigenesis and lung metastasis in murine mammary tumors (Elangovan et al. 2013). Chang et al (2006) demonstrated that induction of Ha-Ras increases promoter methylation of *RECK* (Reversion inducing cysteine rich protein with Kazal motifs) which was reversed by the addition of 5^′^-azacytidine and DNMT3B siRNA indicating Ras induced DNMT3B is primarily responsible for the promoter methylation of *RECK* gene (Chang et al. 2006). Our TCGA analysis showed that 21 missense mutations in *RAS* gene and 3 missense and 9 truncated mutations in *c-JUN*. Interestingly, TCGA analysis showed upregulation of RAS in breast tumor tissues and consistency in stage-wise increase was found (Fig. 5).

#### STAT3-mediated regulation of DNMT isoforms

Signal transducer and activator of transcription 3 (STAT3) belongs to STAT family of transcription factor which upon phosphorylation by the receptor associated tyrosine kinases form homo or heterodimers and translocate into nucleus, where these transcription factors modulate cell proliferation, apoptosis, cell motility, mammary gland involution and angiogenesis (Bromberg and Darnell 2000; Yu et al. 2009). Activation of STAT3 is regulated by phosphorylation at serine and tyrosine residues and post-translationally by the demethylation at K140 and acetylation at K685 (Kang et al. 2015). Constitutive persistent activation of STAT3 has been implicated in the pathogenesis of whole spectrum of malignancies including that of breast tumors (Burke et al. 2001). STAT3 also been shown to increase methylation of CpG islands in genes including *PTPN6*, *ESR1* and *SOCS3 *via upregulating DNMT1 expression binding to the promoter region (Zhang et al. 2005; Thomas 2012; Huang et al. 2016). Lee et al. (2012) showed that acetylation of STAT3 is crucial for promoter methylation of tumor suppressor genes and treatment with resveratrol resulted in demethylation in breast tumor and melanoma cell lines (Lee et al. 2012). This indicated, role of resveratrol as epigenetic modifier in breast tumors. Elevated levels of acetylation of STAT3 at K685 is known in subjects with melanoma, colon carcinoma and triple negative breast cancer compared to respective normal tissues. STAT3 K685 acetylation led to hypermethylation and silenced several genes including *CDKN2, STAT1 and DLEC1* and authors further showed that promoter methylation are not as a consequence of STAT3 phosphorylation at Y705 but due to acetylation (Lee et al. 2012; Thomas 2012). In addition, chromatin immunoprecipitation confirmed the binding of acetylated STAT3 to DNMT1 promoter as a consequence of increased p300 levels and subsequent interaction with DNMT1 in malignant T lymphocytes and breast cancer cell lines (Macaluso et al. 2003; Zhang et al. 2005). TCGA data analysis revealed the significant aberrant expression of both STAT3 and p300 in breast tumor tissues indicating their crucial role in epigenetic changes during tumorigenesis and metastasis (Fig. 5).

#### MDM2-mediated regulation of DNMT isoforms

MDM2 is a nuclear localized E3 ubiquitin ligase and promotes accelerated cell growth and tumor formation upon inducing proteasomal degradation of tumor suppressor proteins such as TP53 and RB (Michael and Oren 2003; Sdek et al. 2005). Overexpression of MDM2 has been observed in several cancer types including breast tumors. Distinct promoter usage and alternative splicing of MDM2 has been reported in breast cancer cell lines and breast tumor tissues leading to aberrant expression of MDM2 disrupting TP53 pathway in breast tumors (Lukas et al. 2001; Okumura et al. 2002). TCGA analysis also confirmed aberrant expression of MDM2 and p53 in breast tumor tissues indicating their role in breast tumor initiation and progression (Fig. 5).

### Transcriptional repression

#### Role of TP53 in regulating DNMT isoforms

*TP53* gene encodes for the tumor suppressor protein containing DNA binding, oligomerization and transcriptional activation domains. In response to cellular stress, TP53 regulates expression of target genes thereby inducing cell cycle arrest, programmed cell death, senescence, DNA repair and metabolic changes (Hager and Gu 2014; Kang et al. 2015; Kruiswijk et al. 2015). Mutations in *TP53* gene are associated with variety of malignancies including Li-Fraumeni syndrome (Petitjean et al. 2007), colon cancer (Munro et al. 2005), lung cancer (Peifer et al. 2012), esophageal cancer (Makino et al. 2010), ovarian cancer (Ahmed et al. 2010), breast cancer (Olivier et al. 2006) and are attributed to aggressiveness of the disease (Schmitt et al. 2002). Miller et al. (2005) have shown that TP53 expression signature is consistently associated with patient survival and is a prognostic and predictive indicator in breast cancer (Miller et al. 2005). The loss of *TP53* gene is often through large deletions, frame shift mutations, however, many mutations in the tumor cells are found to be single nucleotide missense variants leading to dominant negative phenotype of variable degree. Majority of these mutations are localized to DNA-binding domain resulting in loss of transcriptional function of TP53 (Miller et al. 2005). TP53-binding sites have been identified in the 5′ flanking region and exon-1 (− 19 to + 317) of promoter region of the human DNMT1 gene. Several p53-binding regions were also identified in the 5′ region of the mouse DNMT1 (Peterson et al. 2003; Lin et al. 2010b). In MCF-7 cells overexpression of TP53 showed reduced levels of both SP1 and DNMT1. Coimmunoprecipitation assay showed that TP53 does not bind directly to SP1 and instead promoter activity was reduced with mutant SP1-binding site in luciferase reporter assays indicating that DNMT1 expression is regulated by TP53 via SP1 in breast tumor cells (Zhang et al. 2016). Furthermore, in MDA-MB-468, triple negative basal type breast cancer cell line mutant TP53 was shown to stabilize the DNMT1-MeCP2-HDAC1 complex leading to suppression of *ESR1, survivin and cdc25c* gene expression via hypermethylation (Estève et al. 2005; Arabsolghar et al. 2013). Under physiological conditions, p53 repressed transcription of both DNMT3A and DNMT3B while inducing TET1 and TET2 which are crucial for the conversion of 5-methyl cytosine to 5-methyl hydroxy cytosine (Laptenko and Prives 2017). In addition, Wang et al. (2005) showed that interaction of DNMT3A with TP53 is crucial for the stability of DNMT3A and transcriptional suppression of TP53-mediated gene expression in MCF-7 cell lines (Wang et al. 2005). TCGA data analysis revealed that deregulation in TP53 expression is significantly correlated with breast cancer stages which may be due to mutations in *TP53* gene and/or due to the overexpression of MDM2 which targets TP53 to proteasomal degradation. Mutation analysis for *TP53* in TCGA showed 684 missense mutations, 436 truncated mutations, 19 frame shift mutations and 2 fusions have been reported in breast cancer patients (Fig. 5).

#### RB-mediated regulation of DNMT isoforms

*RB* gene encodes for the retinoblastoma (RB) protein which negatively regulates cell cycle progression (Weinberg 1995). The protein maintains the overall integrity of the chromatin structure through the interaction with BRG1 SUV39H1 (Shao and Robbins 1995), SWI/SNF (Zhang et al. 2000) and HDAC1 (Luo et al. 1998). The active dephosphorylated form of the protein binds directly to *E2F1* promoter region and acts as the transcriptional repression of E2F1 targeted genes and when phosphorylated by CDK3/cyclin-C it promotes G0-G1 transition and progression of the cell cycle (Ren and Rollins 2004). Robertson et al. (2000) demonstrated that DNMT1 forms the complex with RB/E2F/HDAC1 and represses transcription of E2F responsive promoters in both in vitro and in vivo using calf brain (Robertson et al. 2000)*.* DNMT1 promoters in prostate epithelial cell line of both mouse and human harbors functional E2F-binding sites which is crucial for the regulation of RB/E2F (McCabe et al. 2005). Disruption of p16^INK4A^, maintain RB in its active form, transcription was associated with aberrant CpG DNA methylation in breast cancer cell lines and primary breast tumors (Herman et al. 1995). Macaluso et al. (2003) showed that pRb2/p130-E2F4/5-HDAC1-DNMT1-SUV39H1 multimeric complex suppressed ERα expression by promoter hypermethylation in breast cancer cell lines (Macaluso et al. 2003). In addition, the same laboratory demonstrated that 5′-Aza-2′-deoxycytidine reorganization of pRB2/DNMT1 multimeric complex on *ERα* gene promoter and modulated its expression in breast cancer cell lines (Macaluso et al. 2007). These data together suggests that RB pathway is crucial for the regulation of DNMT1-mediated gene promoter methylation.

The promoter region of DNMT3A harbors E2F1-binding sites and was transcriptionally repressed by RB/E2F complex formed at these sites. Tang et al. (2012) reported that RB depletion resulted in overexpression of MDM2 leading to transcriptional activation of DNMT3A which subsequently reduced expression of downstream tumor suppressor genes via promoter hypermethylation (Tang et al. 2012). TCGA data analysis revealed that significant alteration in the transcript expression of both RB and E2F can be attributed to mutations in breast tumors- 20 missense mutations, 61 truncated and 2 fusion of *RB*; 12 missense and 7 truncated mutations in E2F1 (Fig. 5).

#### FOXO3A-mediated regulation of DNMT isoforms

Forkhead Box O3A (FOXO3A) belongs to forkhead family of transcription factors characterized by the conserved distinct DNA-binding domain ‘forkhead’ (Benayoun et al. 2011). FOXO family proteins have been considered as tumor suppressors due to their inhibitory action on cell proliferation and inducers of apoptosis (Wang et al. 2014). Ectopic overexpression of FOXO3A upregulated Bcl2 interacting mediator of cell death (BIM) resulted in impaired tumor progression in both in vitro and xenograft models of breast tumors (Zou et al. 2008; Smit et al. 2015). In paclitaxel sensitive breast cancer cell lines, paclitaxel reduced tumor cell survival and induced apoptosis by upregulating BIM via FOXO3A (Sunters et al. 2006). Human primary breast tumors negative for phospho-Akt showed FOXO3A in the cytoplasm and high levels of IκB kinase β-modulator of NFκB pro inflammatory pathway. Over expression of FOXO3A reversed the IκB kinase β dependent stimulation of cell cycle progression, proliferation and tumorigenesis in mice (Hu et al. 2004). Yang et al (2014) showed that FOXO3A binds to promoter region (+ 163- + 173) of *DNMT3B* and negatively regulates promoter activity. FOXO3A nuclear localization reduced the DNMT3B expression by establishing repressed chromatin structure, whereas knockdown of FOXO3A resulted in open chromatin structure and increased DNMT3B mRNA and protein levels (Yang et al. 2014). TCGA analysis showed significantly reduced mRNA expression of FOXO3A in breast tumors which was correlated with stages. Three missense mutations and three fusions are reported in *FOXO3A* gene according to TCGA-BRCA database (Fig. 5).

#### Other transcription factors regulating DNMT isoforms

Several other transcription factors have been reported regulating expression of DNMT isoforms in both physiological and pathological conditions. Homeobox B3 induced DNMT3B overexpression resulted in the epigenetic silencing of tumor suppressor gene RASSF1A in MDA-MB-231 cell lines (Palakurthy et al. 2009). The *t*(8;21) translocation in acute myeloid leukemia induced the formation of RUNX1 (runt-related transcription factor 1)-MTG8 which was shown to interact directly or indirectly with DNMT1 and there by silencing target gene expression (Liu et al. 2005). In breast cancer cells knockdown of RUNX1 resulted in aberrant expression genes related to chromatin organization- NEAT1, MALAT1 and ECM components including fibronectin 1 and fibrillin 2 (Barutcu et al. 2016). In addition, specificity in the expression of transcription factors confined to specific tissue and/or cell type may limit their role in regulating DNA methylation to specific tissues and/or cell types.

The transcription factor screening using TF-binding input showed DNMT isoforms shows redundancy for transcription factors. This indicates the intricate regulation of DNMT isoforms expression (Fig. 4).

## Epigenetic regulation of DNMT isoforms

Next, we screened for CpG density on promoters of genes encoding human DNMT isoforms. Our bioinformatic analysis and literature survey indicates that DNMT isoforms contain CpG islands (Fig. 3). However, the complete regulation of DNMT isoforms expression via their CpG sequence methylation is not well understood.

## Post-transcriptional regulation of DNMT isoforms

### miRNA-mediated regulation of DNMT isoforms

**Fig. 6:**
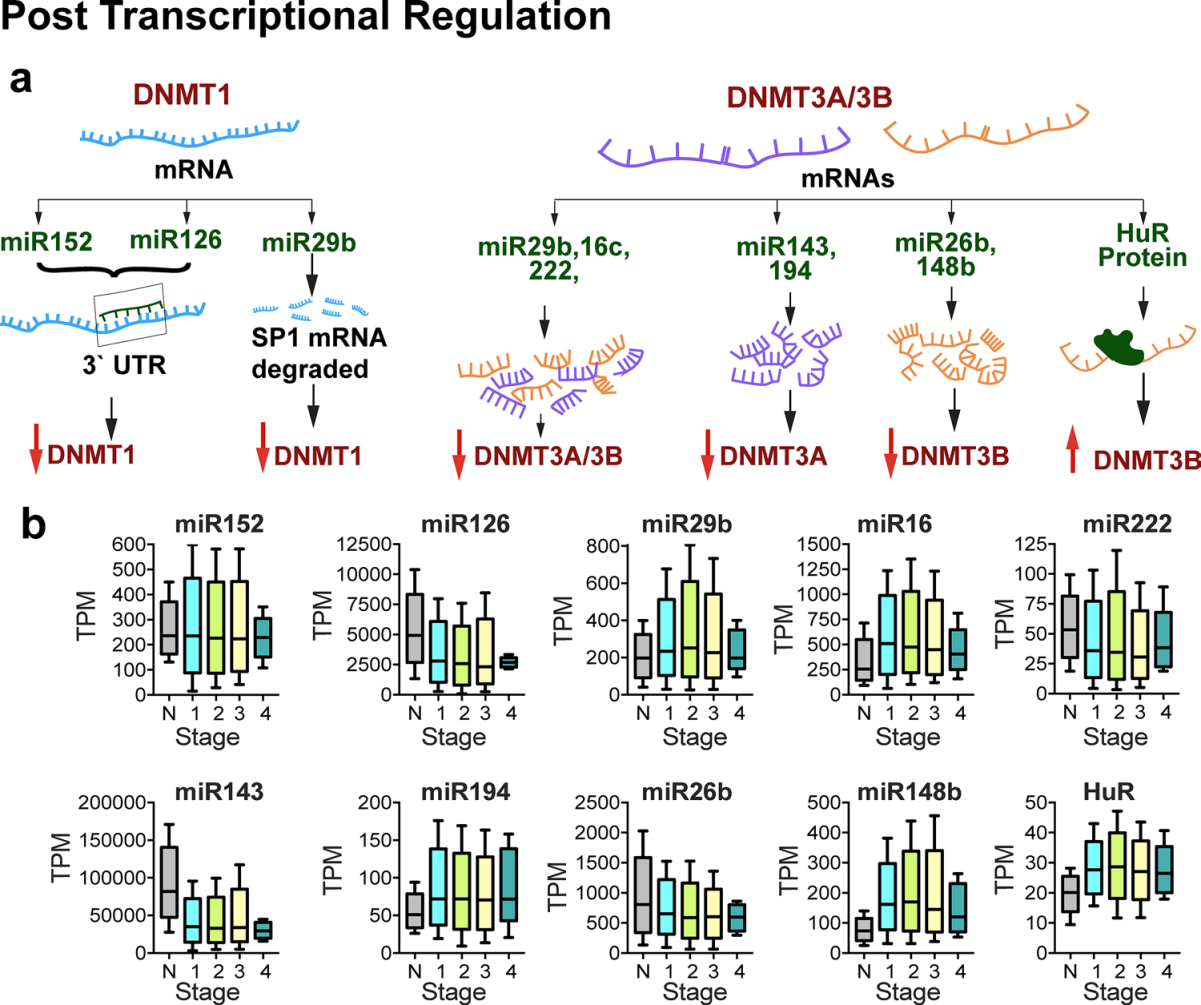
Post-transcriptional regulation of human DNA methyl transferases. **a** miRNAs and HuR protein that target DNMT isoforms are indicated. miRNAs (micro RNAs) bind to either 3′ UTR or coding region destabilizes the mRNA and subsequently protein levels are reduced. HuR (Hu-antigen R) binds to 3′ UTR od DNMT3B and increases its stability. *DNMT* DNA methyl transferase, *miRNA* microRNA, *mRNA* messenger RNA, *UTR* untranslated region. **b** The expression levels of these miRNAs in different stages- normal (*n* = 76), stage 1 (*n* = 135), stage 2 (*n* = 427), stage 3 (*n* = 171), stage 4 (*n* = 8) of breast cancer are shown

Micro RNAs (miRNAs) induce translational repression, deadenylation or degradation by imperfectly aligning with the 3′ UTR region of target mRNAs (Filipowicz et al. 2008). Dysregulation of miRNA expression have been reported in several tumors including lung, bladder, pancreatic, liver, esophageal, colon, prostate, ovarian and breast (Lu et al. 2005; Melo and Esteller 2011; Ferreira and Esteller 2018). Recent studies have indicated that specific miRNAs regulate DNA methylation machinery and are linked to aberrant methylation pattern in altering cancer epigenome (Fig. 6). The expression of miR-29a and miR-29b were found to target DNMT3A and DNMT3B in in vitro and in vivo models of breast cancer (Sandhu et al. 2012). Knock down and re-expression of miRNAs showed that miR-26b, miR-29a, miR-29b, miR-29c and miR-148b down regulate DNMT3B in breast cancer cells (Sandhu et al. 2012). The miR-143 was shown to directly target DNMT3A mRNA and downregulate protein expression in colorectal cancers (Ng et al. 2009) and breast (Ng et al. 2014). Additionally, miR-194 was demonstrated to regulate DNMT3A expression pattern in drug resistant breast cancer cells and DNMT3A was shown to be the direct target of miR-194 (Le et al. 2010).

### Long noncoding RNA-mediated regulation of DNMT isoforms

Long noncoding RNA (lncRNA) is a pivotal factor in regulating chromatin structure, chromosome looping, nucleosome positioning, DNA methylation and histone modifications (Böhmdorfer and Wierzbicki 2015; Ferreira and Esteller 2018). Numerous studies have demonstrated that lncRNA breast cancer growth (Shen et al. 2015), proliferation, invasion (Shi et al. 2015), apoptosis (Tuo et al. 2015) and chemotherapeutic resistance (Li et al. 2015). Furthermore, Wu et al. (2018) demonstrated that linc00152 promotes tumorigenesis of triple negative breast cancer by targeting DNMT1, DNMT3A and DNMT3B, which resulted in modulation of BRCA1 and PTEN expression both in vitro and in vivo. In addition, authors showed that knockdown of lnc00152 in MDA-MB-231 cells resulted in down regulation of DNMT1, DNMT3A and DNMT3B in association with up regulation of BRCA1 and PTEN leading to decreased proliferation, invasion and enhanced apoptosis of these cells (Wu et al. 2018).

### piRNA-mediated regulation

Aberrant expression of piRNAs and piwi family proteins is associated with hall marks of cancer and have shown promise as novel diagnostic and prognostic biomarkers in several malignancies such as lung squamous cell carcinoma, gastric carcinoma, colon adenocarcinoma and breast cancers (Cheng et al. 2011; Mei et al. 2013). Mouse germ cells that were deficient in Piwi subfamily members *Mili* or *Miwi-2* showed defective de novo methylation of transposons (Kuramochi-Miyagawa et al. 2008). Genome wide methylation microarray analysis using HumanMethylation 450 array platform showed MCF cell lines transfected with piRNA mimics showed 117 genes were differentially methylated. Authors validated that mRNA expression of 6 genes -*CDK4, FAM150A, KDM3A, LHX5, SYCE1* and *VAMP3*- were significantly associated with the expression of piRNA (Fu et al. 2014). The direct interaction between piRNA or Piwi family proteins with DNMT3A and DNMT3B have not been explored so far.

### HuR (Hu-Antigen R)-mediated post-transcriptional stabilization of DNMT transcripts

HuR protein is a member of embryonic vision family (ELAV) and possess three RNA recognition motifs rich in AU- and U-rich sequences and binds to target mRNAs with higher specificity and affinity. HuR protein is reported to alter the stability of mRNA or translation or both there by regulating target gene expression (Kuwano et al. 2008). HuR has been shown to play major role in cell proliferation, immune response, stress response, senescence and tumorigenesis post-transcriptionally by influencing the stabilization of mRNAs including those of cyclin A, cyclin B1, cyclin D1, c-fos, c-myc, TNF- α, Mcl1, cyclooxygenase-2, β-catenin, p21, p27, p53, VEGF, iNOS, GM-CSF, SIRT1, uPA and uPAR (Levy et al. 1998; Wang et al. 2000b, a; Brennan and Steitz 2001; Ming et al. 2001; Chen et al. 2002; Sengupta et al. 2003; Tran et al. 2003; Lal et al. 2004; Song et al. 2005; Abdelmohsen et al. 2007, 2008). Bioinformatic analysis showed that DNMT3B mRNA 3′ UTR region has a consensus motif HuR and is one of the putative target of HuR. López de Silanes et al. (2009) experimentally showed that HuR bind to DNMT3B mRNA and enhance its stability leading to increased steady state levels of DNMT3B. Furthermore, the authors demonstrated that cisplatin treatment lower DNMT3B levels via inducing the dissociation of DNMT3B mRNA from HuR followed by instability of mRNA in colorectal carcinoma cell lines (López de Silanes et al. 2009). Recently, CRISPR/Cas9-mediated deletion of RMST (rhabdomyosarcoma 2-associated transcript) in MCF cell lines showed that RMST promotes HuR binding to DNMT3B 3′ UTR region increasing stability of DNMT3B and its upregulation (Peng et al. 2020).

## Post-translational regulation of DNMT isoforms

**Fig. 7:**
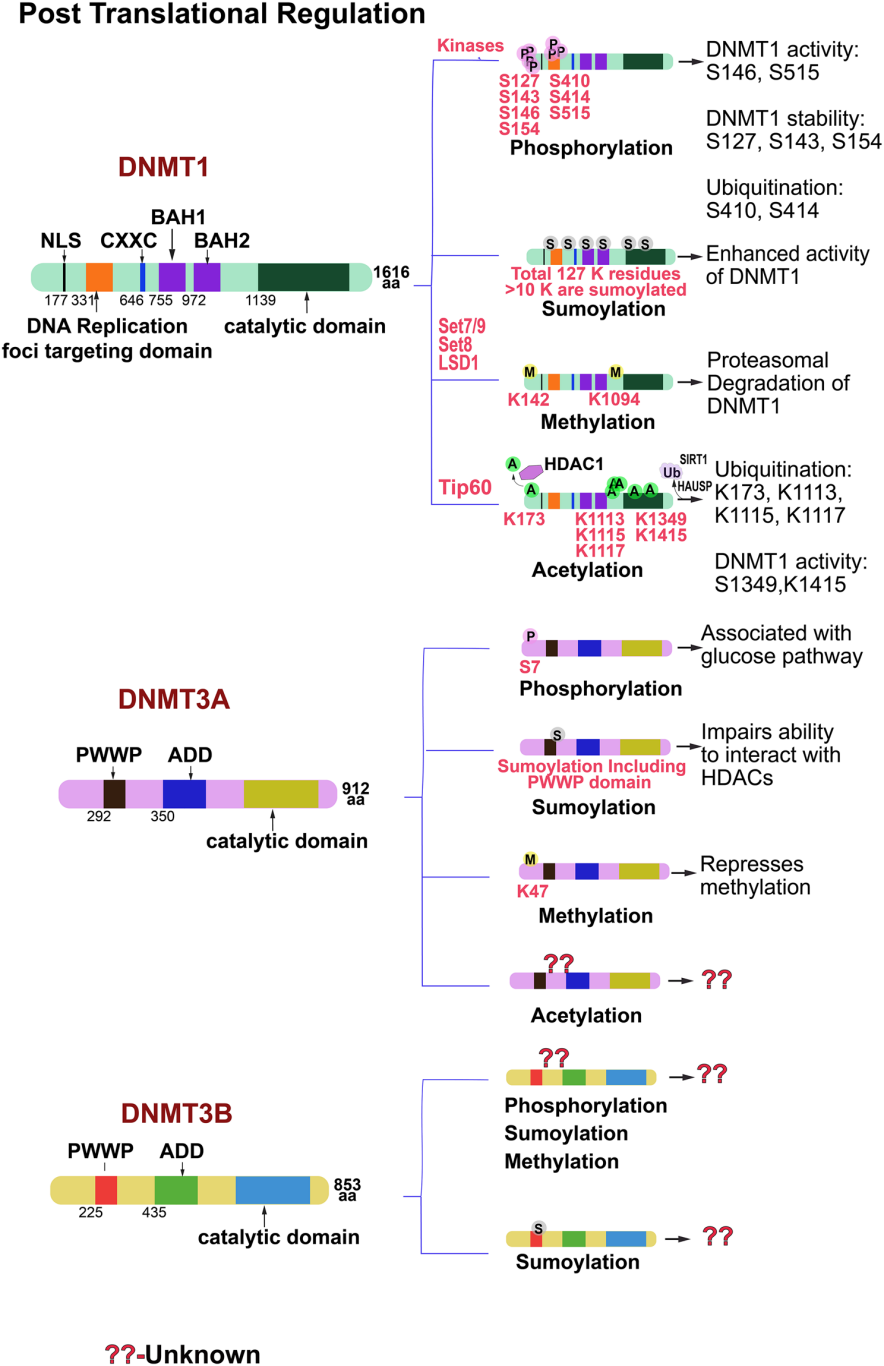
Post-translational regulation of human DNA methyl transferases. Summary of covalent post-translational modifications of DNMT1, DNMT3A and DNMT3B are shown along with the position, amino acid modified and biological significance. These modifications include phosphorylation (P), SUMOylation (S), methylation (M), acetylation (A) and ubiquitination (Ub). The proteins mediate these modifications, if any, are indicated. *DNMT* DNA methyltransferase, *SUMO* small ubiquitin like modifier, *Set7/9* SET domain containing protein 7/9; *Set8* SET domain containing protein 8, *LSD1* Lysine specific demethylase 1, *Tip60* Tat interacting protein 60 kDa; *HDAC* histone deacetylase, *SIRT1* Sirtuin 1, *HAUSP* herpes virus associated ubiquitin specific protease

Post-translational modifications (PTMs) including phosphorylation, acetylation, SUMOylation, glycosylation, ubiquitination, nitrosylation, sulfation, butyrylation, propionylation, ADP-ribosylation, methylation and citrullination of proteins play significant role in regulating gene expression, protein activity and function (Reinders and Sickmann 2007). Biochemical and molecular biology studies have confirmed that stability, catalytic properties and functions of DNMTs are also regulated by phosphorylation, acetylation, methylation, SUMOylation and ubiquitination (Fig. 7).

### Protein phosphorylation

Following the initial identification of insect DNMT1 phosphorylation at S^515^ (Glickman et al. 1997), enumerable phosphorylated serine and threonine residues are identified in purified DNMT1 from human cells by targeted high-throughput proteomic approaches. More than sixty phosphorylation sites have been mapped on human and mouse DNMT1 protein (https://www.phosphosite.org) albeit only few of them have been functionally characterized. The phosphorylated S^515^ located within the amino terminal of replication foci targeting domain is require to preserve the interaction between DNMT1 N-terminal and catalytic domains that is crucial for the enzyme activity (Goyal et al. 2007). Casein kinase 1 delta/epsilon reduces DNA-binding affinity of DNMT1 by phosphorylating S^146^ in the N-terminal regulatory domain (Sugiyama et al. 2010). In mouse and human glial cells, Akt and PKC phosphorylate DNMT1 at S^127^/S^143^ and S^143^ respectively and there by control the interaction of DNMT1 with PCNA and UHRF1 (Hervouet et al. 2010). Phosphorylation of S^143^ residue by Akt1 stabilizes the DNMT1 protein in a cell cycle dependent manner (Estève et al. 2011). Previous studies have shown that Akt inactivates GSK3β (Ser/Thr kinase) resulting in recruitment of E3-ubiquitin ligase βTrCp followed by degradation of downstream target proteins (Sharma et al. 2002; Taketo 2004). Lin et al (2010a, b) demonstrated that GSK3β phosphorylates DNMT1 at S^410^ and S^414^ and induce DNMT1 binding to βTrCp leading to its proteasomal degradation (Lin et al. 2010a). In addition, PKC α, βI, βII, δ, γ, η, ζ and μ phosphorylates DNMT1 and phosphorylation of DNMT1 in its N-terminal domain by PKCζ reduces its methyltransferase activity in vitro. Furthermore, phosphorylation of DNMT1 by CDK 1, 2 and 5 at S^154^ is shown to enhance the protein stability and activity (Hervouet et al. 2010; Lavoie et al. 2011; Lavoie and St-Pierre 2011). Phosphorylation of either single or multiple residues of both serine and threonine reduces the activity of DNMT1 which is shown involved in the regulation of global DNA methylation changes and tumorigenesis in HEK-293 and HeLa cell lines (Lavoie et al. 2011). Using cell lines and extensive bioinformatic analysis, Esteve et al. (2016) showed that 14-3-3 is a reader protein of DNMT1 S^143^ and interact with phosphorylated DNMT1 inducing aberrant DNA methylation and alter gene expression leading to tumor progression and cell invasion in breast cancer (Estève et al. 2016). Phosphorylation of DNMT3 family proteins and their functions are not well understood. However, experimental evidences suggests that Casein kinase 2 phosphorylating DNMT3A in both mice and humans (Deplus et al. 2014; Richter et al. 2019). This phosphorylation guides DNMT3A to specific sequence of the genome and controls subnuclear partitioning (Deplus et al. 2014). Sacco et al (2016) showed that phosphorylation of DNMT3A at S^7^ site is associated with glucose response and regulates target gene expression in human pancreatic beta and HEK 293 cell lines (Sacco et al. 2016).

### SUMOylation

Small ubiquitin like modifier (SUMO) proteins are capable of covalently and reversibly attached to other proteins in cells. SUMOylation has emerged as critical post-translational mechanism regulating protein stability, sub cellular localization, enzyme activity and protein–protein interactions (Verger et al. 2003). DNMT1 possess more than ten putative SUMOylation sites throughout its primary amino acid sequences (Xue et al. 2006). Using in vitro wild type/mutant cell lines and in vivo knockout models, Lee and Muller (2009) demonstrated that SUMOylation of DNMT1 is mediated by SUMO1 and is crucial for methyl transferase activity of DNMT1 (Lee and Muller 2009). Recently, Borgermann et al. (2019) showed that 5-Aza-2′-deoxycytidine treatment targets DNMT1-DNA crosslinks by enhancing SUMOylation of DNMT1 in Human U2OS cell lines. Authors, further showed that inhibition of SUMOylation by the knockdown of SUMO E2 enzyme UBC9 strongly impaired the DNMT1-DNA adducts and DNA replication (Borgermann et al. 2019). This indicated that SUMOylation of DNMT1 plays critical in the resolution of DNMT1-DNA adducts post-replicatively. Although the complete mechanism and enzyme which aid in SUMOylation of DNMT3A and DNMT3B are unidentified. However, preliminary data suggest that SUMOylation of DNMT3A transforms its ability to interact with HDAC (Kang et al. 2001a; Ling et al. 2004). Furthermore, Seo et al. (2019) demonstrated that mutations at R882 residues in acute myeloid leukemia patients resulted in enhanced SUMOylation by SUMO1 protein leading to weak complex formation between DNMT3A and HDAC. This weaker association of DNMT3A-HDAC complex induces acetylation of H3K27 and overexpression of PD-L1 attributing to escape from immune surveillance and drug resistance (Seo et al. 2019).

### Methylation, acetylation and ubiquitination of DNMT isoforms

Lysine methylation is another functionally important reversible post-translational modification of DNMTs. DNMT1 contains over 120 lysine residues and is methylated at multiple sites (Wang et al. 2009). Wang et al. (2009) showed that in DNMT1 K^1096^ (K^1094^ in humans) methylation is regulated by Set7/9 and LSD1 (Lysine specific demethylase 1) and affects global DNA methylation in murine embryonic stem cells (Wang et al. 2009). Parallel research by Esteve et al. (2009), showed that in humans Set7 methylate K^142^ of DNMT1 and knockdown of Set7 led to increased DNMT1 levels (Estève et al. 2009). Studies have shown that methylation at K^142^ is inhibited by Akt1-mediated phosphorylation of DNMT1 at S^143^ and the methylation of DNMT1 at K^142^ is recognized by CRL4 ubiquitin E3 ligase to target DNMT1 for ubiquitin dependent proteasomal degradation (Estève et al. 2011; Leng et al. 2018). Set8 is also found to regulate DNA methylation targeting methylated DNMT1 and methylated UHRF1 to proteasomal degradation which is an opposite action to LSD1-mediated DNMT1 protection (Zhang et al. 2019). Methylation-dependent DNMT1 proteolysis is tightly coordinated with cell cycle regulation in that activity of DNMT1 being highest in S phase. During S phase of the cell cycle, DNMT1 was protected by LSD1 and PHF20L1 via inhibiting the binding of L3MBTL3 to DNMT1. Upon dissociation from PHF20L1 and reduced LSD1, L3MBTL3 is known to bind to methylated DNMT1 leading to proteolysis of DNMT1 in late S and G2 phases (Leng et al. 2018). Furthermore, L3MBTL3- CRL4 complex is also shown to induce proteolysis of methylated E2F1. DNMT1 along with E2F1 forms complex with HDAC1 and RB53 to regulate target gene expression and degradation of both methylated E2F1 and methylated DNMT1 by L3MBTL3- CRL4 complex indicate their highly controlled regulation during cell cycle (Leng et al. 2018; Levy 2019).

G9a (also known as euchromatin histone methyl transferase)-mediated demethylation of DNMT3A at K^47^ is demonstrated to be recognized by the chromodomain of methyl-H3K9-binding protein MPP8 (M phase phospho protein) forming DNMT3A/H3K9/MPP8 complex which represses de novo methylation. G9a is shown to methylate DNMT1 at K^70^ but the functional consequences are yet to be determined (Chang et al. 2011).

Agoston et al (2005) demonstrated that deletion of N-terminal 120 amino acids mapped to destruction domain of DNMT1, which is responsible for proper ubiquitination, results in increased protein stability and genomic cytosine hypermethylation in normal human breast epithelial cells (Agoston et al. 2005). Authors further showed that this destruction domain is dysfunctional in MCF-7 breast cancer cell lines compared to normal human breast epithelial cells and is responsible for differential expression of DNMT1 among these cell lines (Agoston et al. 2005). Furthermore, Zhou et al (2008) showed that deletion of 120 amino acids of N-terminal region inhibits LBH589 (clinically relevant HDAC inhibitor)-induced DNMT1 ubiquitination in MDA-MB-231 cells, indicating that impairment in regular ubiquitination leads to genomic hypermethylation in breast cancer cell lines (Zhou et al. 2008).

DNMT isoforms have been shown to destabilize by acetylation. An acetyltransferase Tip60 along with RGS6 and DAMP1 are shown to promote acetylation of DNMT1 at K^173^, K^1113^, K^1115^, K^1117^ and subsequently leads to ubiquitination by E3 ligase UHRF1 followed by proteasomal degradation during late S phase. Conversely, HAUSP (herpesvirus-associated ubiquitin specific protease) and HDAC1 protected DNMT1 from proteolysis via deubiquitination and deacetylation respectively (Du et al. 2010). In contrast, SIRT1 is shown to physically interact with DNMT1 and deacetylates DNMT1 both in vitro and in vivo. Deacetylation at K^1349^ and K^1415^ residues of DNMT1 by SIRT1 has been shown to enhance the methyl transferase activity of enzyme in breast cancer cell lines (Peng et al. 2011). Using the extensive proteomics analysis 12 new acetylated lysine residues have been identified in DNMT1 both in vitro and in vivo and the deacetylation impaired methylase activity and transcription repression (Peng et al. 2011; Kar et al. 2012).

### Regulation of enzyme activity of DNMT isoforms

#### Auto inhibitory mechanism and allosteric regulation

The autoinhibitory mechanism for methylation has been potential target for novel small molecule inhibitors for cancer therapy. Several SAM and DNA competitors including RG108 (Siedlecki et al. 2006; Asgatay et al. 2014), RG119-1 (Rondelet et al. 2017), SGI-1027 (Datta et al. 2009), CM-272 (San José-Enériz et al. 2017), BIX-01924 (Rotili et al. 2014), DC-05 and DC-517 (Chen et al. 2014) have been shown to acts as demethylating agents and antiproliferative in various human malignancies. Recently, Muvarak et al. (2016) showed that Poly (ADP-ribose) polymerase inhibitors enhanced binding of PARP1 and DNMT1 at the DNA damage site inducing cytotoxic effects in the breast cancer xenograft model (Muvarak et al. 2016). Furthermore, using molecular simulation, Xie et al. (2019) demonstrated that SFG (DNMT1 and DNMT3A inhibitor), DG-05 (selective inhibitor of DNMT1) and GSKex1 (selective inhibitor of DNMT3A) inhibitors binds specifically to SAM-binding pocket in particular Val1580/Trp893, Asn1578/Arg891adn Met1169/Val1665 of DNMT1/DNMT3A via van der Waals interaction and inhibit the methylation (Xie et al. 2019). Recently, Krishna et al. (2017) demonstrated that small molecule DNMT1 inhibitors JFD01881, RJC02836, RJC02837 and 5-azacytidine binds to Cys1226 and Glu1266 within SAM-binding pocket and inhibit methylation activity. Authors further showed that these compounds display significant in vitro anti-proliferative activity using MDA-MB-231 breast cancer cell lines (Krishna et al. 2017).

### Regulation of DNMTs by interacting factors

**Fig. 8:**
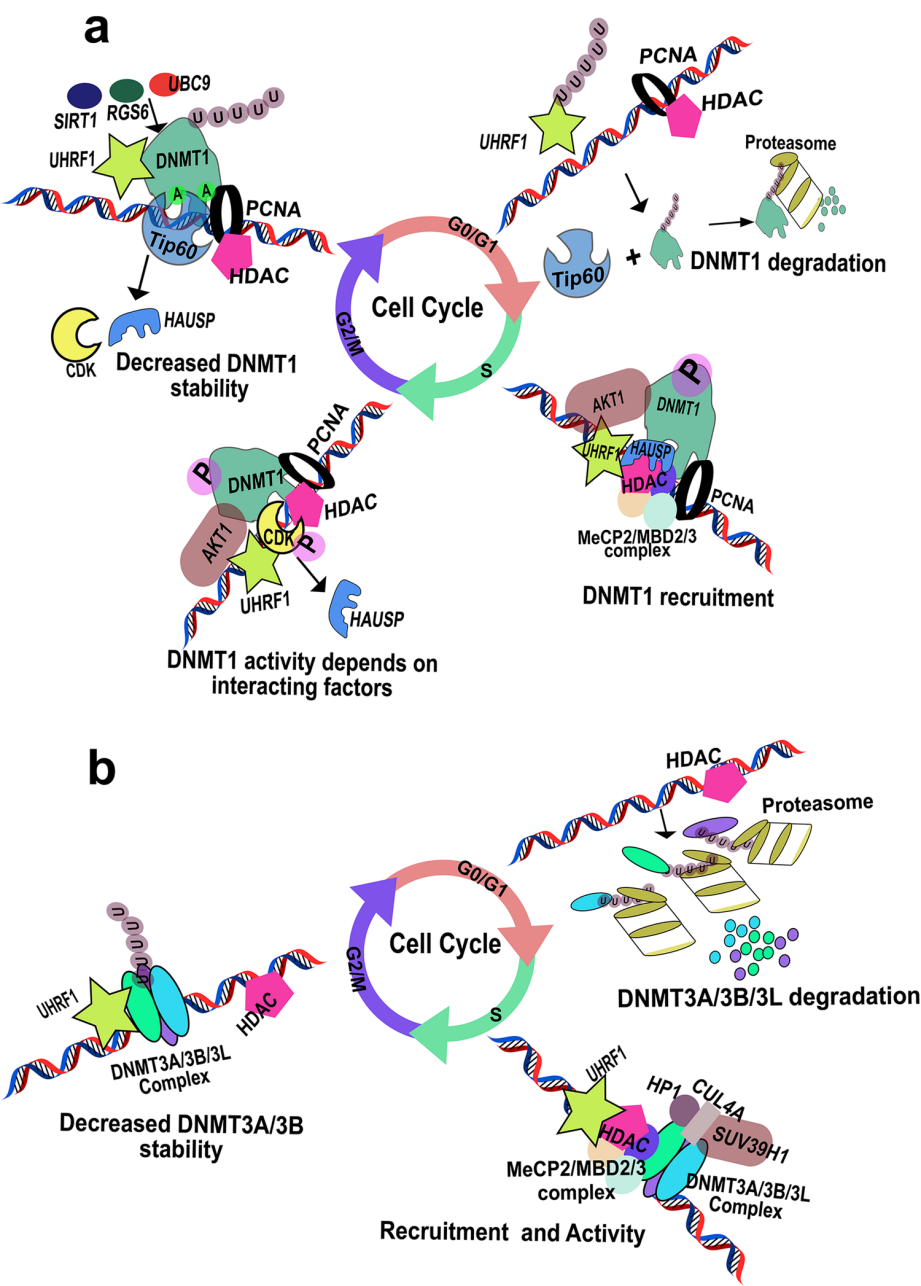
Regulation of DNMT isoforms by interacting factors: Regulation of DNMT1 is cell cycle dependent. The recruitment of DNMT1 to replication site is carried out by MeCP2, MBD2/3. The interaction of DNMT1 with HAUSP, HDAC1/2 and PCNA increases the stability and activity of DNMT1. Furthermore, interaction of DNMT1 with AKT1 and CDK/cyclin increases methylation activity. At the G2/M phase acetylation, SUMOylation followed by ubiquitination be various interacting factors including Tip60, UHRF1, RGS6, SIRT1 and UBC9 leads to decrease in DNMT1 stability and proteasomal degradation (**a**). Similarly, DNMT3A/3B/3L multimeric complex was shown to be recruited by CUL4A/HP1/SUV39H1 complex and the stability was increased by the interaction with MeCP2/MBD2/3 and HDAC. Stability of DNMT3 multimeric complex was shown to be decreased due to the ubiquitination and the complex was later cleared by proteasomal degradation

Over the years, mounting evidences have reported that the large variety of proteins interact with DNMT isoforms including methyltransferases (both DNA and histone), DNA/chromatin-binding proteins, chromatin modifiers, tumor suppressors, transcriptional activators and cell cycle regulators (Hervouet et al. 2018). These interactions subsequently results in stimulation or inhibition of enzyme activity, increase or decrease the efficiency of the enzyme, guide DNMT isoforms to methylation sites, enable dissociation from target DNA or specific methylation patterns maintenance. The discrepancy between the high processivity of replication (1 nucleotide per ~ 0.035 s) and low methylation turnover rate (70–450 s per methyl group addition) by recombinant DNMT1 in vitro (Jackson and Pombo 1998; Pradhan et al. 1999) suggests that additional mechanisms and proteins are required for interacting machineries to increase the DNMT isoforms activity normal physiological processes (Fig. 8).

DNMT1 carries out post-replicative conservation of methylation patterns encompassing complete genome in successive generation by directly interacting with PCNA. The non-obligatory interaction of DNMT1 and PCNA enhances the efficiency of methylation activity by two fold and aids for diverse enzyme kinetics in faithful propagation of methylation information (Iida et al. 2002). DNMT1 has been shown to bind to DNMT3A and DNMT3B suggesting an intricate network between DNMT isoforms for the efficient and non-erroneous methylation of target DNA (Kim et al. 2002). Although DNMT1 is self-capable to recognize and bind hemi methylated CpG sites, interaction with methylated CpG-binding proteins such as MeCP2, UHRF family and MBD2/3 have shown to increase the enzyme efficiency. MeCP2 induces chromatin compaction by binding to DNA and interact with DNMT1 via TRD domain. MeCP2 and MBD2/3 recognizes methylated CpG sites and MBD3 binds to HDAC1 and HDAC2 which ultimately interact with DNMT1 (Tatematsu et al. 2000; Robertson et al. 2000; Kimura and Shiota 2003; Bronner et al. 2007). These interactions suggest the complex mechanism in maintaining hypomethylation and transcriptional repression. This complex also interacts with DMAP1 (DNMT associated protein 1) and transcriptional coregulator DAXX (death domain associated protein) mediating repression which is independent of HDAC (Muromoto et al. 2004). In addition, these interactions enhance the heterochromatin region formation at highly methylated regions. Recent studies have shown that UHRF1 is essential for DNA methylation maintenance and genetic aberration in UHRF1 leads to hypomethylation which was similar to homozygous knock down in embryonic stem cells (Bostick et al. 2007). Throughout the S-phase co-localization and interaction of UHRF1 and DNMT1 leads to preferential binding of these proteins to hemimethylated DNA sequence along with H3K9me3 (Arita et al. 2008). The crystal structure of SET and Ring associated (SRA) domain of UHRF1 in complex with hemimethylated DNA revealed that the 5-methyl cytosine is flipped out of the DNA double helix suggesting DNMT1 preferably not directly bind to hemimethylated DNA rather DNMT1 is recruited by UHRF1 (Avvakumov et al. 2008; Hashimoto et al. 2008; Qian et al. 2008). Studies showed that UHRF2, another protein of UHRF family also interact with DNMT1 and represses epigenetic changes indicating the non-redundant functions of UHRF1 and UHRF2 during the development (Pichler et al. 2011). Nishiyama et al. (2020) demonstrated that replication timing-dependent dual mono ubiquitination of PCNA associated factor 15 (PAF15) via UHRF1 is a prerequisite for chromatin recruitment of DNMT1 and for high fidelity DNA methylation inheritance in mouse embryonic stem cells (Nishiyama et al. 2020). Moreover, UHRF1, UHRF2 and DNMT1 together interact with DNMT3A and DNMT3B exhibiting the complex interplay in establishing methylation patterns and its maintenance (Fatemi et al. 2002; Pichler et al. 2011). In addition, Liu et al (2018) showed that trimethylation of H3K27 by EZH2 leading to the formation of H3K27me3-EZH2-DNMT1 complex formation and hypermethylation of Kibra (wwc1) gene CpG island resulted in epithelial mesenchymal transition of triple breast cancer cell lines (Liu et al. 2018). Besides these, DNMT1 association with transcription factors and regulators such as CFP1 (Butler et al. 2008), SP1 (Estève et al. 2007), SP3 (Estève et al. 2007), STAT3 (Zhang et al. 2005) and NRIP1 (Kiskinis et al. 2007) aid in the regulation of cell signaling.

On contrary to DNMT1, which is mainly recruited in replication foci during *S*-pahse of cell cycle, DNMT3A and DNMT3B are not concentrated to these foci. During the replication process, DNMT3B interact with human chromosome associated proteins (hCAP) C, E and G and condensing complexes leading to chromosomal condensation indicating DNMT3B dependent methylation is, at least partially independent of DNA replication (Margot et al. 2001; Geiman et al. 2004). DNMT3A/DNMT3B interactions with DNMT3L has been demonstrated in recruiting DNMT3A and DNMT3B on DNA during genomic imprinting. The DNMT3L and DNMT3A forms either dimer or tetramers which results to refolding of DNMT3A leading to increased de novo enzyme activity up to 20-fold (Suetake et al. 2004; Kareta et al. 2006). The recruitment of DNMT3A/DNMT3L complexes was more frequent on Alu sequences imprinted gene promoters and CpG-rich regions (Jia et al. 2007; Glass et al. 2009). Furthermore, interaction of DNMT3 isoforms with LSH (lymphoid specific helicase) increased the processivity of these enzymes and nullification of LSH in embryonic stem cells provoked hypomethylation of repeat elements and decreased expression of specific genes (Myant and Stancheva 2008). DNA de novo methylation is initiated by SUV39H1 and subsequent binding of HP1 leads to recruitment of DNMT3A and/or DNMT3B on the target sequence (Fuks et al. 2003). DNMT3B interaction with SUV39H1 is involved in the pericentric heterochromatin methylation and not in the methylation of centromeric regions. On the other hand interaction of DNMT3B with CENP-C favors methylation of centromeric areas (Gopalakrishnan et al. 2009).

These DNMTs interacting factors were reported to be altered in tumors. In the CUL4A (component of cullin-ring-based E3 ubiquitin protein ligase complex) over expressing tissues such as hepatomas and breast cancer DNMT3B activity was enhanced due to its interaction with CUL4A-NEDD8 resulting in hypermethylation (Shamay et al. 2010). Jin et al. (2010) reported that UHRF1 is overexpressed in BRCA1 hyper methylated breast tumor tissues and overexpression of UHRF1 in breast cancer cell lines led to deacetylation of H3/H4 followed by DNMT1 recruitment on to *BRCA1* promoter and hypermethylation (Jin et al. 2010). Dong et al. (2013) reported that elevation in Snail-SUV39H1 complex was in coordination with the elevation in H3K9me3 at the E-cadherin promoter leading to the recruitment of DNMT1 and gene silencing causing enhanced epithelial mesenchymal transition, a function of DNMT1 which is entirely different from the previously known biological function, in basal like breast cancer cell lines (Dong et al. 2013). Furthermore, Duvall-Noelle et al. (2016) showed that LASP-1 (LIM and SH3 protein 1) interaction with UHRF1-DNMT1-Snail1 complex is associated with alteration in epigenetic modifications leading to breast tumor cell migration, local invasion and metastasis (Duvall-Noelle et al. 2016). Recently, Pradhan et al. (2019) demonstrated that the treatment of breast cancer cell lines with the sublethal dosage of hydrogen peroxide induces DNMT1, Snail, Slug and HDAC1 via ERK pathway and induces chromatin remodeling at the E-cadherin promoter. Authors showed that treatment of breast cancer cell lines with 5-aza-deoxycytidine prevented the promoter CpG methylation of E-cadherin and treatment of cells with ERK inhibitor reduced the expression of DNMT1, Slug and snail indicating the synergistic role of histone methylation, deacetylation and methylation-mediated chromatin remodeling during breast tumorigenesis (Pradhan et al. 2019). Table 1 summarizes alterations of interacting factors of the DNMT isoforms in breast tumor tissues as analyzed in TCGA database.

**Table 1:** List of proteins interacting directly or indirectly with DNMTs and their expression in breast cancer based on TCGA database

Protein	Full name	Function	Levels in breast cancer (TCGA)
Chromatin modifiers			
HDAC1	Histone deacetylase 1	Deacetylation of N-terminal residues on histones	
HDAC2	Histone deacetylase 2	Deacetylation of N-terminal residues on histones	
LSD1	Lysine specific demethylase 1	Demethylate lysine residues of histone	
LSH	Lymphoid specific helicase	DNA strand separation	
G9a (EHMT2)	Euchromatic histone lysine methyltransferase 2	specifically mon- and di- methylates H3K9	
SUV39H1	Suppressor of variegation 3–9 homolog 1	Histone methyltransferase specifically trimethylates H3K9	
EZH2	Enhancer of zeste 2 polycomb repressive complex 2 subunit	Polycomb group protein Catalytic subunit of PRC2/EED/EZH2 complex	
SETD7	SET domain containing 7, lysine methyltransferase	Histone methyltransferase specifically monomethylates H3K9	
KAT5	Lysine acetyltransferase 5	Catalytical subunit of histone acetyl transferase complex	
TIP5	TTF-I interacting peptide 5	Essential component of nuclear remodeling complex	
SMARCA4 &5	SWI/SNF Related, Matrix Associated, Actin Dependent Regulator Of Chromatin, Subfamily A, Member 4 & 5	Helicase that possess intrinsic ATP-dependent nucleosome remodeling activity	
HP1 β	Heterochromatin protein 1 beta	Recognizes and binds methylated H3K9	
Transcription regulators			
DMAP1	DNMT associated protein 1	Involved in transcriptional repression/activation	
CFP1	CXXC finger protein 1	Transcriptional activator, Exhibits DNA-binding activity specific for unmethylated CpG sites	
NRIP1	Nuclear receptor interacting protein 1	Modulates transcriptional activation/repression	
SNAIL1	Snail family transcription repressor 1	Transcriptional repressor	
Slug	Snail family transcription repressor 2	Transcriptional repressor	
(Methyl) CpG binding			
MeCP2	Methyl CpG-binding protein 2	Methylated CpG-binding protein	
MBD2	Methyl CpG domain-binding protein 2	Methylated CpG-binding protein	
MBD3	Methyl CpG domain-binding protein 3	Methylated CpG-binding protein	
UHRF1	Ubiquitin like PHD and RING finger domain 1	Hemimethylated CpG-binding protein	
UHRF2	Ubiquitin like PHD and RING finger domain 2	Hemimethylated CpG-binding protein, E3 ubiquitin protein ligase	
Cell cycle regulators			
PCNA	Proliferating cell nuclear antigen	Targtes DNMT1 to replication foci	
CENP-C	Centromere protein C	Component of kinetochore plate and cell cycle regulator	
Others			
DAXX	Death domain associated protein	Adapter protein in the MBD2/DAXX/USP7 complex	
DNMT3L	DNA methyltransferase 3 like	Stimulates DNMT3A/3B	
LASP1	LIM and Sh3 protein 1	Cytoskeletal remodeling	
CUL4A	Cullin 4A	DNA damage response and repair	
USP7	Ubiquitin specific peptidase 7	Hydrolase and ubiquitinates target proteins	
UBC9	Ubiquitin carrier protein 9	E2 ubiquitin conjugating enzyme	
AKT1	AKT serine/threonine kinase 1	Ser/Thr protein kinase	
PARP1	Poly (ADP-ribose) polymerase 1	Poly(ADP) ribosylation	
CK1	Casein kinase 1	Phosphorylates large number of proteins	

## Hormonal regulation of DNMT isoforms

**Fig. 9:**
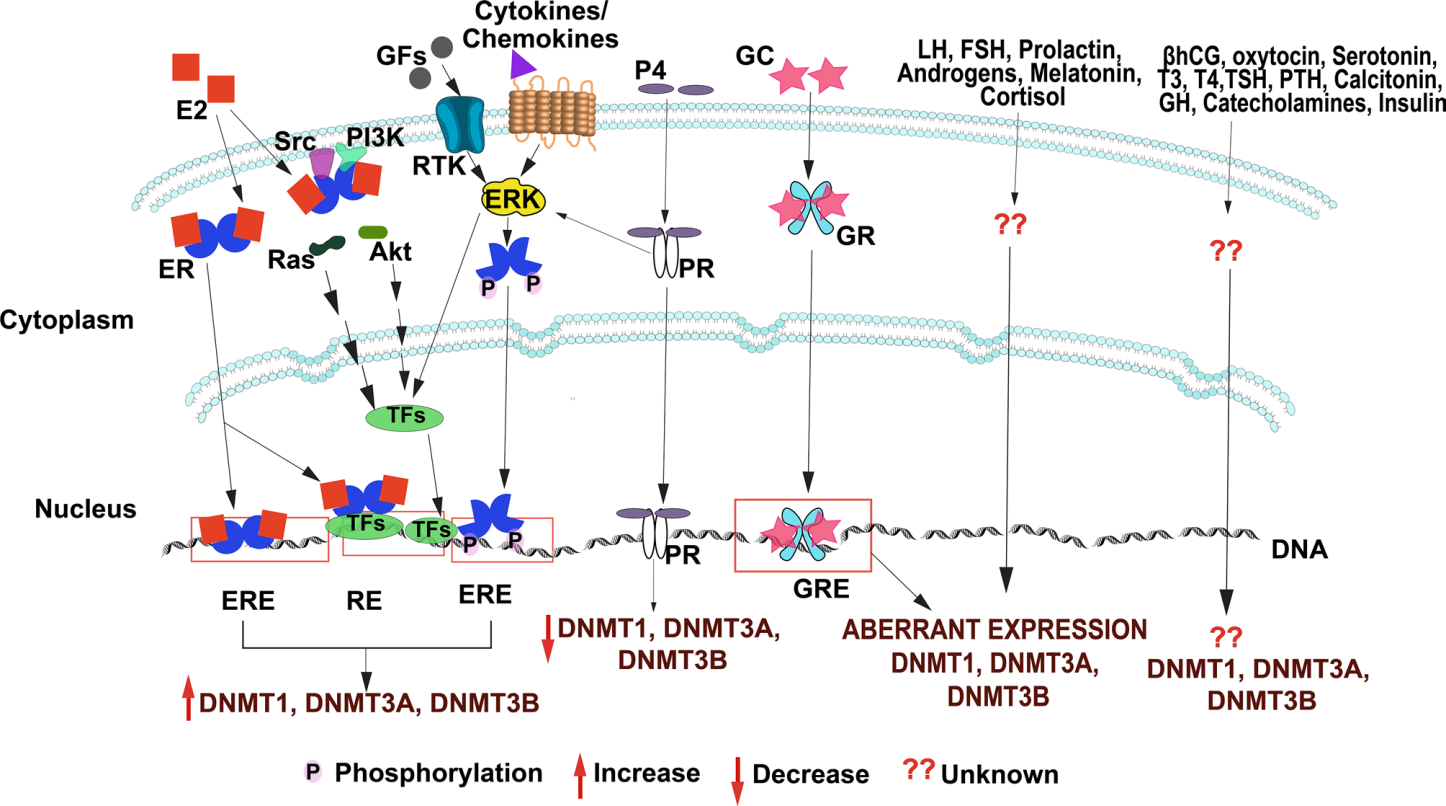
Hormonal regulation of human DNA methyl transferases. Expression of DNMT isoformsin mammary gland in response to different hormones is shown. Estrogen (E2) upon binding to estrogen receptor either directly binds to estrogen response elements on DNA or via activating transcription factors such as AP1and STAT3. Growth factors, cytokines and chemokines activates ERK which in turn phosphorylates estrogen receptor independent of estrogen leading to aberrant DNMT isoforms expression. Progesterone binding to its receptor directly binds to DNA or activates ERK and subsequently activates transcription factors leading to decreased expression of DNMT isoforms. DNMT isoforms also showed to possess binding sites to glucocorticoid receptor and expression of mRNA is influenced by glucocorticoids. On the other hand, gonadotropins such as LH and FSH, prolactin, androgens, melatonin and cortisol shown to induce aberrant expression of DNMT1, DNMT3A and DNMT3B when present, however, the pathway through which they act is unknow. Other hormones which significantly influence breast cancer growth, such as βhCG, oxytocin, Serotonin, T3, T4, TSH, PTH, Calcitonin, growth hormone, Catecholamines and insulin have not yet been shown whether or not they affect expression of DNMT isoforms. *E2* estrogen, *ER* estrogen receptor, *ERE* estrogen response element, *RE* response element, *Src* steroid receptor coactivator, *PI3K* Phosphatidylionosiyol-3-kinase, *TFs* transcription factors, *P4* progesterone, *PR* progesterone receptor, *GC* glucocorticoid, *GR* glucocorticoid receptor, *GRE* glucocorticoid response element

Hormones are essential for the growth, function, maintenance of tissue homeostasis of mammary gland and variation in the levels are known to cause several pathological conditions. The mammary gland undergoes several major changes postnatally in every stages of woman’s life including puberty, pregnancy, lactation and involution. Initially at birth, mammary gland consists of a primary duct and few secondary ducts and resembles that of man. During puberty, dramatic changes occur including significant development of ducts in terms of elongation, branching and accumulation of fat in the adipose tissue of the breast in females. Along with every ovarian cycle, mammary gland undergoes cyclic changes and major differentiation with lobuloalveolar growth occurs from pregnancy to throughout lactation. Post-lactation involution of mammary gland results in regressed ducts and lobuloalveolar structures. In all these stages of mammary gland hormones play major role at the genetic, molecular and epigenetic levels (Rijnkels et al. 2010; Brisken and O’Malley 2010; Macias and Hinck 2012; Holliday et al. 2018). In the gynecological malignancies such as breast carcinoma, ovarian cancers and endometrial adenocarcinoma involvement of hormones and their receptors in the tumor initiation, growth, invasion and metastasis have been reported (Garrett and Quinn 2008; Rice 2010). The role of hormones (if any) in regulating DNMTs in the breast cancer context is discussed below and illustrated in Fig. 9.

### Estrogen

Epidemiological data suggests that women are at 100 fold higher risk of breast cancer development than men and bilateral oophorectomy before the age 35 years reduces 75% of life time breast cancer incidence (Santen et al. 2007). Enhanced period of exposure to estrogen due to early menarche, late menopause, obesity and high bone density is shown to be associated with the increased risk of breast cancer (Hsieh et al. 1990). Clinical studies have shown that women with high plasma free estradiol levels experience 2.5 fold higher rate of breast cancer over the years than those who have low plasma free estradiol levels (Kaaks et al. 2005; Beattie et al. 2006). Lowering the estrogen by tamoxifen or raloxifene treatment reduced breast cancer incidence by 38% and aromatase inhibitors reduces it by 50–65% in the high risk women (Cuzick et al. 2003). Furthermore, during adjuvant therapy use of aromatase inhibitors or anti-estrogens showed to prevent the development of cancer occurrence in contralateral breast (Fisher et al. 1998; Santen et al. 2010). Effects of estrogen on target cells in the breast are mediated via several mechanisms (Fig. 9). Most widely accepted mechanism utilizes estrogen receptor-mediated transactivation of genes which favor cell proliferation and survival (Liao et al. 2014; Jameera Begam et al. 2017). Another mechanism elucidates genotoxicity of by-products of estrogen metabolism directly damage DNA altering apoptosis, DNA repair and cell cycle regulations resulting in clonal expansion of pre-cancer cells (Lippert et al. 2003). Cheng et al (2008), for the first time showed that exposure to estrogen altered DNA methylation patterns in humans (Cheng et al. 2008) and subsequently Kovalchuk et al. (2007) showed that estrogen induced epigenetic changes occur prior to the tumor initiation in mice models (Kovalchuk et al. 2007). In vitro studies have shown that estradiol treatment increased DNMT1, DNMT3A and DNMT3B levels, activity, binding to the target genes and methylation (Wu et al. 2019). Furthermore, estrogen receptor alpha (ERα) was shown to interact directly with DNMT1 and DNMT3B and recruit them on the target genome to suppress the gene expression (Si et al. 2016). Our bioinformatic analysis revealed that DNMT1, DNMT3A and DNMT3B promoters harbors multiple ERα-binding sites (Fig. 4).

### Progesterone

Progesterone acts as pro-proliferative factor for the breast tissues and functions in concert with estrogen and estrogen receptors to induce the expansion of glandular structures during puberty (Brisken and O’Malley 2010). Due to the mode of action and functions, progesterone and progesterone receptors gained constant attention for their emerging role as critical modulators of gynecological cancers including breast cancer (Diep et al. 2015). Progesterone induces proliferation of breast cancer cells by activating Ras/ERK pathway (Migliaccio et al. 1998). The elevated progesterone levels combined with estrogen levels has shown more detrimental effects on breast by guiding cells towards tumorigenesis than either of them alone (Hankinson et al. 2004). Though the progesterone play key role in breast tumor development its effect on DNA methylation in breast is not studied. The ER^+^/PR^+^ breast cancer cells have shown differential methylation pattern than ER^−^/PR^−^ breast cancer cells (Li et al. 2010a; Verde et al. 2018). Furthermore, studies have demonstrated that progesterone receptor regulates methylation and expression of ESR1 (ERα) upon binding to ESR1 promoter sequence (Verde et al. 2018). In addition, progesterone treatment either alone or combined with estrogen showed to downregulate DNMT1, DNMT3A and DNMT3B levels leading to hypomethylation in human endometrial stromal cells (Yamagata et al. 2009). The authors also showed that varied cyclic levels of these hormones during luteal phase and mid secretory phase were associated with differential levels of DNMT isoforms in endometrium (Yamagata et al. 2009).

### Gonadotrophins

Gonadotrophin releasing hormone antagonists, that suppress the release of FSH (Follicle Stimulating Hormone) and LH (Luteinizing Hormone), have been shown to be effective in the treatment of breast cancer in fertile women (Robertson and Blamey 2003). Planeix et al (2015) demonstrated that VEGFR2 negative endothelial cells of breast cancer expressed FSHR and these FSHR positive blood vessels extended 2 mm to 5 mm outside the tumor periphery indicating their involvement in vascular remodeling in anti VEGFR2 resistance breast tumors (Planeix et al. 2015). Sanchez et al (2016), showed that the FSHR and LHR are functionally expressed in several breast cancer cell lines and the extent of expression was found to be involved in the modulation of cell migration and invasion via activation of G proteins on the plasma membrane (Sanchez et al. 2016). Furthermore, the inclusion of LH or FSH in the cancer cell growth medium in vitro phosphorylates moesin (actin remodeling protein) and focal adhesion kinase ultimately leading to the formation of molecular bridges between integrins, focal adhesion complexes and actin which enhances cell motility (Sanchez et al. 2016). Uysal et al (2018), showed that administration of FSH and/or LH analogues caused aberrant expression of DNMT1, DNMT3A, DNMT3B and DNMT3L and also affected their subcellular localization in mouse oocytes and embryos (Uysal et al. 2018). LH surge has been demonstrated to hypomethylate the promoter regions of several genes including CYPA11a1 and CYPA19a1 which are involved in the estrogen and progesterone synthesis (Okada et al. 2016) In addition, gonadotropin surge can causes change in methylation pattern indirectly by controlling estrogen and progesterone levels (Okada et al. 2016).

### Pregnancy associated hormones

Upregulation of pregnancy associated hormones such as estrogen, progesterone and others are shown to be responsible for transiently increased risk for breast malignancies during pregnancy and post-partum period. Placental production of estrogen, progesterone, placental growth factor, human chorionic gonadotrophin and placental lactogen leading to substantial alteration in the hormonal milieu during pregnancy which influence the proliferation, growth, differentiation and expansion of mammary gland tissues (Ishida et al. 1992; Smith et al. 2001; Cnattingius et al. 2005; Froehlich et al. 2019). However, these hormones act beneficial in certain circumstances, for instance, ER^+^/PR^+^ MCF or T47D breast cancer cell lines co-cultured with first trimester placental tissue showed reduction of breast cancer cell numbers and reduced expression of ERα on these cells which is responsible for proliferation (Tartakover-Matalon et al. 2010). Furthermore, expression of ERβ, antagonist for tumor cell proliferation and invasion was found to be two fold higher in parous women than in nulliparous women (Asztalos et al. 2010). An important hormone in maintaining pregnancy, human chorionic gonadotrophin, is found to mammary gland protective and reduces the risk of breast malignancies (Russo and Russo 2011). Placental hCG along with tumor suppressor inhibin downregulates ERα expression by inducing CpG methylation (Russo and Russo 2007). However, the ectopic expression of β-hCG in breast cancer patients has shown to be associated with high grade tumors and poor prognosis (Chang et al. 2005). Ectopically expressed β-hCG exerts anti-apoptotic effect by blocking TGFβ receptors, promotes invasion and migration by down regulating E-cadherin, inducing ERK1/2 and MMP-2 (Wu and Walker 2006; Li et al. 2013b, c). Although the direct link between β-hCG and DNMT isoforms have not been established, the increased β-hCG has shown to be associated with global DNA hypomethylation in the DNA isolated from the serum of pregnant women compared to non-pregnant women (Pauwels et al. 2016).

### Glucocorticoids

Glucocorticoids are involved in the development of mammary gland during puberty and pregnancy (Casey and Plaut 2007). The expression of GR is observed in normal breast and all stages of breast cancer tissue with the decline in expression from normal to precancerous lesions and to malignant carcinomas (Teulings and van Gilse 1977; Allegra et al. 1978). Glucocorticoids exerts anti-proliferative and anti-apoptotic activity on breast cancer epithelial cells, at least in part, via modulating transcriptional regulation of genes encoding cell survival pathways such as SGK1 and MKP1/DUSP1 (Mikosz et al. 2001; Wu et al. 2004; Melhem et al. 2009). Furthermore, using triple negative breast cancer mouse xenograft models, Skor et al. (2013) showed that treatment with GR antagonists might be useful in multidrug resistant triple negative GR^+^ breast cancer cells (Skor et al. 2013). However, recently Obradovik et al. (2019) demonstrated that GR activity is higher in metastatic breast tumor and higher expression of GR induces lung metastasis in mouse xenograft models (Obradović et al. 2019). However, there are no studies related to influence of glucocorticoids on DNMT isoforms. Our bioinformatic analysis showed that *DNMT1* promoter harbor putative-binding site for GR (Fig. 4).

### Cortisol

The negative impact of increased stress on human health has been well explained and known to increase the possibilities of developing migraines, diabetes, heart attacks, ulcers and malignancies (Cohen et al. 2012). In accordance with this greater than 70% of breast cancer patients showed high levels of serum cortisol levels. Nineteen years follow up survey of 18,932 women conducted by Nilsen et al. (2008) showed women working in highly fast paced jobs with high stress are more prone to develop breast cancer than the women working at slower pace with less stress (Nielsen et al. 2008). The dysregulated cortisol release showed positive correlation with disease progression, increased mortality rate, recurrence and increased fatigue (Sephton et al. 2000; Abercrombie et al. 2004). The role of cortisol in breast epigenetics has not been established. However, Intabli et al (2019) showed that treatment of triple negative breast cancer cell lines MDA-MB-231 and Hs578T with cortisol decreased the expression of DNMT1 leading to hypomethylation of promoter regions of key tumor suppressor genes including DAPK1, AKT1, ABL1, CDKN1A and MGMT (Intabli et al. 2019).

Oxytocin, prolactin, gonadotrophins, androgens, melatonin, serotonin, thyroid and parathyroid hormones, calcitonin, and catecholamines have been shown to participate in etiology of breast tumor etiology and progression. However, their influence on regulation of DNMT isoforms is poorly understood.

## Influence of cytokines and growth factors in regulating DNMT isoforms

Inflammation has been attributed as one of the hallmarks of cancers and altered levels of cytokines has been shown to regulate global DNA methylation changes in breast cancer (Fleischer et al. 2014; Fogel et al. 2017). Numerous cytokines have been shown associated with chronic inflammation designated as tumor enabling characteristic drive pathogenic changes in breast tumor microenvironment (Esquivel-Velázquez et al. 2014). However, understanding of the involvement of these cytokines in epigenetic modulation in breast cancers is sparse. As mentioned earlier, activated STAT3 which is a downstream signaling molecule for several cytokines belong to IL-6 cytokine family including IL-6, transcriptionally activates DNMT1 leading to hypermethylation of anti-apoptotic genes. IL-6 via IL-6R/STAT3 pathway regulates DNMT1 expression in tumor cells (Huang et al. 2016). Our recent studies in clinically characterized human breast tumor tissues, we demonstrated IL-6 induced proteasomal degradation of DNMT1 which led to promoter DNA hypomethylation of VEGFR2 promoter and subsequently to disorganized sprout formation in endothelial cells isolated from malignant part of breast tissue (Hegde et al. 2020).

Growth factors such as epidermal growth factor, fibroblast growth factor, vascular endothelial growth factor, insulin like growth factor1 and 2 are known to be proliferative to breast cells and are positively correlated with disease progression, end stage, metastatic spread, poor diagnosis and mortality (Richard et al. 1987; Adams et al. 2000; Dickson et al. 2000; Zhang and Yee 2000). Among these, only IGF 1 has been shown to regulate DNA methylation. IGF 1 binding to IGF 1R leads to downregulation of miR152 which elevates DNMT1 levels and also by activating Akt and subsequent nuclear translocation of GSK3 leading to prevention of proteasomal degradation of DNMT1 in breast tumors**.** This results in overall changes in the methylation pattern of cells in vivo (Wen et al. 2017)*.* Breast cancer cells which express human epidermal growth receptor 2 (HER 2) which is activated mainly by epidermal growth factor has shown differential methylation patterns than those breast cells which do not express these receptors (Fiegl et al. 2006). However, underlying mechanisms are unknown.

## Nutrition and diet influencing expression of DNMT isoforms and significance in breast cancers

Breast cancers are complex multi-genic disorders and gene-nutrient interactions has been shown as major contributor in health management and disease prevention (Freudenheim et al. 1996; Franceschi et al. 1996; Rock and Demark-Wahnefried 2002). Over the years, studies in the context of diet and nutrition have shown that nutrient drive epigenetic changes to alter gene expression, susceptibility to disease including cancer (Anderson et al. 2012; Singh et al. 2014; Andreescu et al. 2018). Many studies indicate that early life nutrition exert imprinting effects on genome which might influence the risk of developing multifactorial chronic diseases in the adulthood (Junien 2006; Dolinoy et al. 2007). Accumulating evidences suggests that dietary intake of nutrition alter expression of genes involved in cell cycle regulation, apoptosis and tumor suppressor genes (Landis‐Piwowar et al. 2008; Li and Tollefsbol 2010).

S-adenosyl methionine (SAM) is a methyl group donor in methylation reactions catalyzed by DNMT isoforms (Feil and Fraga 2012). SAM is synthesized from dietary precursors such as methionine (essential amino acid), folate, choline and betaine. Reduced availability of these dietary nutrients results in reduction of SAM synthesis leading to DNA methylation changes, while increased availability of methyl donors showed enhanced methylation reactions. Further availability of nutrients involved in one carbon metabolism such as folate, cobalamin, riboflavin, pyridoxin and methionine have been demonstrated to alter cancer related DNA methylation (Cheng and Blumenthal 2008; Zeisel 2009; Niculescu and Lupu 2011). Low intakes of cobalamin, riboflavin, niacin, pyridoxine and methionine positively correlated with an increased risk for breast cancer and supplementation of folic acid showed reduced breast cancer risk in premenopausal women (Maruti et al. 2009).

Phytoestrogens, including resveratrol and genistein are known to interact with estrogen receptors and regulate estrogen-responsive genes (Thanos et al. 2006). Genistein is a isoflavone from soybean demonstrated to alter DNA methylation of several genes such as p21, p16^INK4A^, c-MYC and BMI1 thereby preventing growth of breast cancer cells (Li et al. 2013a). In addition, Xie et al (2014) showed that genistein decreases DNMT1 expression, methyltransferase activity and global DNA methylation in MCF-7 and MDA-MB-231 cell lines (Xie et al. 2014). Our earlier studies in lab showed genistein induced reduction in PEPCK-C expression is via promoter DNA methylation at cytosine + 34, + 45 and + 71 positions in fibroblasts and contrarily, genistein increased expression of PEPCK-C in HepG2 cell lines (Seenappa et al. 2016). We also demonstrated that genistein maintain glucose homeostasis by inducing glycogenolysis in HepG2 cell lines (Seenappa et al. 2016). Bioflavonoids such as catechins of tea and polyphenols of coffee, curcumin (component of turmeric powder), lycopene found in tomatoes, papayas, watermelons and carrots have shown to alter DNA methylation patterns in various normal and cancer cell lines.

## Viral infections regulating DNMT isoforms

The involvement of viruses and the viral oncogenes in regulating DNMTs has been described earlier in several tumors including that of breast (Hattori and Ushijima 2016). Epstein Barr virus (EBV) have been shown to be involved in the etiology of various malignancies including head and neck cancers, T-cell lymphoma, Burkitt’s lymphoma, gastric carcinoma and breast cancer (Amarante and Watanabe 2009; Tempera and Lieberman 2014). Tsai et al. (2002) demonstrated that introduction of EBV product LMP1 (latent membrane protein 1) oncoprotein in to MCF-7 breast cancer cell line activated DNMT1, DNMT3A and DNMT3B resulted in the silencing of *CDH1* (Tsai et al. 2002). Human immunodeficiency virus type I induces DNMT1 through the response element in the − 1634 to + 71 region leading to the hypermethylation of p16^INK4A^. Huschtscha et al. (2001) showed that normal human mammary epithelial cells can be immortalized by SV-40 induced transformation (Huschtscha et al. 2001). Furthermore, Hachana et al. (2009) showed that methylation of TIMP3, RASSF1A, SHP1 and BRCA1 were higher in case of patients with SV40 positive than matched normal breast tissues indicating the role of virus in breast cancer progression (Hachana et al. 2009).

## DNMT isoforms as therapeutic targets in breast tumors

With the mounting evidence of how DNA methylation orchestrates abnormal gene expression to drive breast tumorigenesis, there is an increasing focus on developing pharmacological interventions for clinical management. Currently two DNMT inhibitors (DNMTi): 5-azacytidine (Vidaza, Celgene) and 5-aza-2^′^-deoxycytidine or decitabine (Dacogene, Supergen) have been approved by US Food and Drug Administration (FDA) for the treatment of acute myeloid leukemia and high risk myelodysplastic syndrome respectively (Kaminskas et al. 2005a, b). Phase I and II clinical trials investigating the efficacy of demethylating agents in breast cancer yielded promising results (Connolly et al. 2017). Triple negative breast cancers which do not express ER, PR or HER2 receptors are not amenable to conventional therapies. Li et al (2010a, b) demonstrated that treatment of breast cancer cell lines with DNMT inhibitors induced epigenetic reactivation of endogenous estrogen and progesterone receptors (Li et al. 2010b). Furthermore, clinical phase II trials conducted by Connolly et al. (2017) showed that improved efficacy of the treatment when 5-azacytidine was administered along with the hormonal therapy (Connolly et al. 2017). Yu et al (2018) showed that decitabine treatment significantly decreases DNMT protein levels and inhibits tumor growth in triple negative breast cancer xenograft models (Yu et al. 2018). This indicated levels of DNMT isoforms might serve as prognostic marker in triple negative breast cancer patients. The list of DNMT inhibitors along with their outcome in clinical management of breast cancers is given in Table 2.

**Table 2:** List DNMT inhibitors in various phases of clinical trials for the treatment of breast cancer

Class of DNMT inhibitors	Compound	Biological effect	References
Nucleoside analogs	5-azacytidine	DNA demethylation, restoration of tumor suppressors, inhibition of tumor growth, phase I clinical trial in combination with valproic acid showed global hypomethylation in patients with advanced breast cancer	Braiteh et al. (2008)
	5-aza-2′-deoxycytidine	DNA demethylation, restoration of tumor suppressors, inhibition of tumor growth, preclinical studies revealed improved outcome when treated along with hormonal therapy	Mirza et al. (2010)
	Zebularine	Zebularine in combination with trichostatin A sensitized breast adenocarcinoma cells to apoptosis	Kong et al. (2019)
	Guadecitabine (SGI-110)	Coadministration of guadecitabine with talazoparib (Poly ADP-ribose polymerase inhibitor) decreased tumor burden and increased overall survival in breast cancer xenograft models	Pulliam et al. (2018)
	5-fluoro-2′-deoxycytidine	Phase I clinical trial revealed that administration of 5-fluoro-2′-deoxycytidine with tetrahydrouridine showed reduced tumor growth in metastatic breast cancer patients	Newman et al. (2015)
	5-azacytidine-5′-elaidate (CP-4200)	Low dependence on nucleoside transporters and improved drug uptake, increased epigenetic potential, increased anti-proliferative activity on breast cance cell lines	Brueckner et al. (2010)
	Arabinofuranosyl-5-azacytosine (Fazarabine)	Significant anti-proliferative effect in vitro; However, no significant effect in phase II clinical trials in several solid tumors metastatic breast cancer patients	Walters et al. (1992), Casper et al. (1992), Ben-Baruch et al. (1993)
	5,6-Dihydro-5-azacytidine (DHAC)	Re-sensitized estrogen refractory breast cancer cell lines to hormonal therapy	Izbicka et al. (1999)
	2^′^-deoxy-N4-[2-(4-nitrophenyl) ethoxycarbonyl]-5-azacytidine (NPEOC-DAC)	Decreased global DNA methylation in MCF-7 breast cancer cell lines	Byun et al. (2008)
Non-nucleoside analogs	Procarine and Procainamide	Specific inhibitor to DNMT1, demethylation and re-expression of *RARβ2* and *p16* genes in breast cancer cells	Segura-Pacheco et al. (2003)
	RG108	In combination with suramin, RG108 induced re-expression and activation of PKD1 leading to inhibition of triple negative breast cancer (TNBC) cell growth	Borges et al. (2014)
	Hydralazine	Combination of hydralazine and thiazolidinedione showed apoptosis and growth arrest of TNBC cells; Phase II trials of hydralazine along with magnesium and valproate showed no significant effectiveness against breast cancer	Candelaria et al. (2007), Jiang et al. (2011)
	Disulfiram	Inhibit tumor entrained endothelial cell migration, modulate ROS accumulation and overcome synergistic resistance to cisplatin in breast cancer cell lines; suppresses stemness and STAT3 signaling in TNBC cell lines	Han et al. (2015), Yang et al. (2019)
siRNA	MG98	Re-expression of estrogen receptor in TNBC cells	Yan et al. (2003)
Natural inhibitors	Curcumin	Showed anti-proliferative effect and induced apoptosis in breast cancer cells; effectively decreased tumor burden in breast cancer xenograft models; Curcumin in combination with hydroxytyrosol and omega 3 fatty acids led to decreased inflammation and pain in post-menopausal breast cancer patients	Huang et al. (1997), Ströfer et al. (2011), Sinha et al. (2012)
	Tea phycophenols (e.g., Epigallocatechin-3-gallate)	Decreased cell viability and proliferation of breast cancer cell lines	Bimonte et al. (2019)
	Genistein	Transcription suppression of hTERT by promoter hypomethylation and subsequently telomerase inhibition in breast cancer cell lines	Li et al. (2009)

## Conclusion

The regulation of gene expression, activity and recruitment of DNMT isoforms have been tightly regulated by the coordinated functions of transcriptional, post-transcriptional, translational and post-translational events. Several extrinsic and intrinsic factors such as hormones, growth factors, cytokines, vitamins and life style/nutrients have been demonstrated to modulate DNMT isoforms in health and disease. From the previous studies and our bioinformatic analysis confirm that alteration in any of these might lead to tumor initiation, aggressiveness, metastasis and differential response to drugs. Interestingly, alterations in DNMT expression and function have also been attributed in prognosis of breast cancer subjects. The knowledge of the complete regulation of DNMT isoforms is inadequate. Although number of drugs have been explored against DNMT isoforms in several malignancies clinically, failure to reverse methylation changes and/or preventing further changes in gene expression indicates the necessity and importance of understanding the regulation of expressions of these proteins.
